# Diffusion-augmented YOLO26-Swin cascaded framework with hybrid SHAP-CAM for autonomous power grid inspection

**DOI:** 10.1007/s43684-026-00135-2

**Published:** 2026-06-09

**Authors:** Stefano Frizzo Stefenon, João Pedro Matos-Carvalho, Viviana Cocco Mariani, Leandro dos Santos Coelho, Kin-Choong Yow

**Affiliations:** 1 Instituto Superior de Engenharia de Lisboa, Instituto Politécnico de Lisboa, Rua Conselheiro Emídio Navarro 1, 1959-007 Lisboa, Portugal; 2 Faculty of Engineering and Applied Sciences, University of Regina, Regina, Saskatchewan S4S 0A2 Canada; 3 LASIGE, Departamento de Informática, Faculdade de Ciências, Universidade de Lisboa, 1749–016, Lisboa, Portugal; 4 COPELABS, Lusófona University, Campo Grande 376, 1749-024 Lisbon, Portugal; 5 Center of Technology and Systems (UNINOVA-CTS) and LASI, 2829-516 Caparica, Portugal; 6 Department of Electrical Engineering, Federal University of Parana, Curitiba, PR Brazil; 7 Graduate Program in Mechanical Engineering, Federal University of Parana, Curitiba, PR Brazil; 8 Graduate Program in Electrical Engineering, Federal University of Parana, Curitiba, PR Brazil

**Keywords:** Bayesian optimization, Diffusion models, Generative artificial intelligence, Explainable artificial intelligence

## Abstract

Deep learning-based autonomous inspection of power grid insulators is challenged by data imbalance and model opacity. This paper presents an end-to-end solution integrating advanced data synthesis, detection, classification, and explainability. First, a conditional diffusion model generates realistic synthetic fault images to balance the dataset. A two-stage architecture based on You Only Look Once version 26 (YOLO26) extra-large and Shifted windows (Swin)-V2-B, called YOLO26-Swin, fine-tuned with Bayesian optimization, performs robust insulator detection and then fault classification. Finally, a novel SHapley Additive exPlanations with Class Activation Mapping (SHAP-CAM) method provides intuitive visual explanations for model predictions. Extensive experiments validate our framework’s superiority: it achieves an F1-score of 0.98149 and a mean Average Precision (mAP)@[0.5] of 0.98951, exceeding leading detection and classification models. This work highlights the efficacy of diffusion models for data augmentation in critical infrastructure and advances the interpretability of vision-based inspection systems.

## Introduction

The electricity distribution power system has the function of supplying electricity to consumers continuously and stably [1]. To improve the reliability of the power grid, inspections are performed to detect broken insulators and flashovers, indicating that the insulation is compromised and the insulator should be replaced [2]. Inspections can be conducted from the ground or aerially, using aircraft photographs. The use of Unmanned Aerial Vehicles (UAVs) allows the inspection of the electricity network in difficult-to-access locations, being a solution that is currently being used by electricity utilities [3].

The use of an image database to classify insulator defects allows the use of models based on Deep Learning (DL) [4], such as Convolutional Neural Networks (CNNs), which are increasingly being used in the field of computer vision [5]. Hybrid models are outstanding in these applications, and can be applied in an aggregated way, such as object detection and image classification, even outperforming state-of-the-art models. Despite the growing use of DL-based models, there are challenges in their application, such as unbalanced data or non-generalist images [6].

These challenges, particularly the small data constraint, mean that models become overfitted to specific conditions and cannot generalize effectively to classes with fewer samples. Although overfitting can be mitigated by using the early stop criterion, the models may not be generalizable to new samples [7]. The use of synthetic images can be a potential solution to this issue. Generative Adversarial Networks (GANs) stand out for generating synthetic images as a solution for data augmentation and mitigating the problem of unbalanced classes [8].

Diffusion models can be used to generate synthetic images for insulator data augmentation. Diffusion models may be more stable during training, reducing the likelihood of generating unrealistic images compared to GANs [9]. Unlike GANs, which can suffer from mode collapse, diffusion models provide better data distribution coverage, ensuring a more diverse set of generated images. Diffusion models can be guided during the generation process using classifier guidance, allowing controlled image synthesis based on specific attributes or descriptions [10].

Given the challenges of detecting faulty insulators, this paper proposes an optimized cascaded framework that detects and classifies insulators separately. For the detection of insulators, the You Only Look Once (YOLO) version 26 extra-large (YOLO26x) is adopted [11]. This model is tuned by Bayesian optimization based on Gaussian process to ensure adequate hyperparameter values. For the classification task, the dataset containing the bounding boxes is augmented with a conditional diffusion model. Considering this augmented dataset, the Shifted windows (Swin)-V2-B model is used for classification.

This paper has the following contributions:

- To ensure the best hyperparameter setup, the proposed approach employs Bayesian optimization for hyperparameter tuning, ensuring that the detection model operates at its best for insulator identification.
- The dataset augmentation with a conditional diffusion proved better than using a conditional Wasserstein GAN to create synthetic images or the use of only original images.
- The SHapley Additive exPlanations (SHAP) merged with the Class Activation Map (CAM), creating a hybrid structure to provide better explainable results. SHAP-CAM assists operators in decision-making regarding electrical power grid inspections.
- For the insulator detection, the proposed method (hypertuned YOLO26x) outperforms the DEtection TRansformer (DETR), Faster Region-based CNN (Faster R-CNN), RetinaNet, Single-Shot Detector (SSD), YOLOv10 to YOLO26, and modified YOLO models (Spatial Transformer Networks YOLO (STN-YOLO) and Squeeze-and-Excitation YOLO (SAE-YOLO)).
- For the classification, the Swin-V2-B model overcomes the Visual Geometry Group (VGG), Residual neural Network (ResNet), EfficientNet, Vision Transformer (ViT), and other Swin models.

The next sections of this work are organized as follows: Section 2 presents work related to fault identification using object detection methods to improve electrical system inspection. Section 3 explains the hybrid method proposed in this paper. In Section 4, the setup used to carry out the comparisons, the evaluation metrics, and the data set considered are presented. Section 5 focuses on presenting and discussing the results, comparing the proposed method with other state-of-the-art models. Finally, Section 6 presents this paper’s conclusion and suggestions for future work. The acronyms used in this work are presented in Table 1.

**Table 1:** List of acronyms

Acronym	Full form
Adam	Adaptive moment estimation
AdamW	Adam with Weight decay
CAM	Class Activation Map
CNN	Convolutional Neural Network
cGAN	Conditional GAN
DL	Deep Learning
DETR	DEtection TRansformer
GAN	Generative Adversarial Network
GenAI	Generative Artificial Intelligence
GFLOPs	Giga FLoating-point Operations Per second
GLA	Global-Local Attention
GP	Gaussian Process
HiResCAM	High-resolution CAM
IoU	Intersection over Union
MCI	Multiscale Channel Information
mAP	Mean Average Precision
ML	Machine Learning
ReLU	Rectified Linear Unit
ResNet	Residual neural Network
R-CNN	Region-based CNN
RMSprop	Root Mean Square propagation
SAE-YOLO	Squeeze-and-Excitation YOLO
ScoreCAM	Score-weighted CAM
SGD	Stochastic Gradient Descent
SHAP	SHapley Additive exPlanations
SSD	Single Shot Detector
STN-YOLO	Spatial Transformer Networks YOLO
Swin	Hierarchical vision transformers using Shifted windows
UAV	Unmanned Aerial Vehicle
ViT	Vision Transformer
VGG	Visual Geometry Group
XAI	EXplainable Artificial Intelligence
XGradCAM	EXtended Gradient-weighted CAM
YOLO	You Only Look Once

## Related works

This section presents an overview of previously conducted studies on insulator defect detection, continuous monitoring, and inspection of insulators for preventing failures in the electrical power grids using YOLO-based models and augmented datasets.

### Overview of YOLO applications

Manual inspections have high operational costs, due to the large geographic areas with adverse weather conditions [12]. In this context, automated insulator detection from aerial images using YOLO serves as the foundational step toward real-time condition assessment and classification, enabling the deployment of UAVs [13]. With the increasing use of UAV systems, insulator defect detection using Machine Learning (ML) and deep neural networks has arisen as a promising and viable solution [14].

Progress has been made in using object detection methods in the power system sector during the last few years, focusing on insulator fault identification and monitoring [15], where YOLO has been frequently used for anomaly detection [16], especially to check the health of insulators. Several versions of YOLO have been modified over the years. The first version was proposed in 2015 by Redmon et al. [17], and Redmon et al. [18], called YOLO or YOLOv1, and currently, in 2025, there is YOLOv12 proposed by Tian et al. [19]. Unlike traditional methods that analyze an image several times in different regions, YOLO processes it in a single pass through the model, making it fast and efficient [20]. Currently, YOLO26 presented by Sapkota et al. [21] is available for similar object detection tasks.

Each version of the YOLO model includes multiple scaled variants, with suffixes indicating differences in model size and complexity [22]. Specifically, *n* denotes the nano version, *s* represents small, *m* corresponds to medium, *l* stands for large, and *x* represents extra-large. Additional suffixes include *t* for tiny, *c* for compact, *b* for balanced, and *e* for extended. These variations provide a range of trade-offs between classification performance and inference speed, enabling the choice of the most appropriate model based on computational constraints and application requirements [23].

Stefenon et al. [24] proposed a hybrid approach for the detection and classification of faulty insulators, integrating a customized YOLOv5u for insulator detection, evaluated against other YOLO variants, with the optimized Quasi-ProtoPNet architecture for classification. The model is assessed using six different CNNs baseline architectures. Considering an F1-score equal to 0.95165, the optimized hybrid model using DenseNet-161 stood out in the comparisons to VGG-16, VGG-19, ResNet-34, ResNet-152, and DenseNet-121.

Using a synthetic dataset and an upgraded YOLOv5 model, Song et al. [25] proposed a fault detection method for transmission line components using the convolutional block attention module to raise detection performance. Comparative analysis shows that, in terms of performance, the proposed approach outperforms other commonly used object detection networks. Sadykova et al. [26] evaluated insulator images with the presence of ice, snow, and water using different YOLO models. Insulator fault detection using the coordinate attention mechanism and feature channel shuffle operation, YOLO was evaluated by Cao et al. [27].

By decreasing feature redundancy and increasing the inference speed of the feature extraction network, the ghost convolution based on YOLOv5s has been introduced to improve precision and recall in detecting insulator flaws [28]. The YOLO-S model was proposed by Yi et al. [29] and compared with YOLOv5s for insulator defect detection. In the proposed YOLO-S, the S-Intersection over Union (IoU) loss function replaced the original CIoU loss function. The Sigmoid-weighted Linear Unit (SiLU) function was replaced by Mish, HardSwish, and a rectified linear unit, among other replacements. The results were promising when compared to existing detection algorithms under the same conditions.

The cross-modality information attention YOLO model was used by Liu et al. [30] to enhance the insulator defect detection capabilities. A meteorological domain synthesis module was built to translate weather-conditioned insulator images into the real domain. Their proposed model outperformed the state-of-the-art UAV-based multi-domain insulator tasks. Stefenon et al. [31] using 240 inspection photographs of power distribution systems, proposed the hypertuned YOLO model, with hyperparameters optimized by a genetic algorithm, to classify insulating components in good condition, in need of repair, or in need of replacement. The performance results were promising considering the results of the eigenvector-based CAM. The eigenvector-based CAM was integrated into the model to provide an explainable framework [32].

Wang et al. [33] proposed a Multiscale Channel Information (MCI) with Global-Local Attention (GLA) for YOLO. While GLA captures local spatial details and global context information, MCI fully extracts and uses multiscale feature map information, improving the network’s capacity for learning. According to experimental findings, the MCI-GLA increases the average performance of the YOLOv4 to YOLOv8 models in identifying insulator breakdown defects. Li et al. [34] presented another study considering the multiscale attention alignment adversarial domain adaptation YOLO, integrating domain adaptation and generalization. The results proved that their model significantly improved the performance of insulator defect detection in previously unseen complex cloudy domain scenarios.

He et al. [35] proposed an improved YOLOv8 for detecting multiple insulator conditions, such as normal, self-explosive, damaged, and flashover. The designed model exhibited high detection performance. Jing et al. [36] also considered the YOLOv8 to improve the feature extraction capability to recognize defective insulators from transmission lines. Li et al. [37] considered the wise-IoU to optimize detection performance. Their method applies ConvNeXt, a bi-directional feature pyramid network, wise-IoU, and small object detection heads. The model incorporates multi-scale feature fusion and small object detection into YOLOv8 to improve the performance of identifying diverse equipment under varying scales, partial obstruction, and real-time operational constraints

Li et al. [38] presented a modified YOLOv8 model to detect insulator defects in complex backgrounds. The wise-IoU loss function was added to reduce the negative impact of low-quality pictures, and a C2f network was built by integrating the convolutional receptive field coordination attention module. A cross-validation scheme was used to ensure that the subsets completed training and testing, thus minimizing generalization errors. Insulator data with four fault categories (normal, faulty, dirty, and aged) were tested with high precision.

The results from the YOLOv8 network were fused with frequency domain features selected using the extreme gradient boosting algorithm by Wang and Cheng [39]. They applied this method for fast load identification with generalization capability, achieving an identification performance of over 99%. Zhang et al. [40] suggested another model for detecting insulator defects that considers the different backgrounds of power transmission lines. Experiments demonstrated the performance of the model in both general and complex scenery.

To recognize insulators and detect faults, Li et al. [41] proposed a modified hybrid attention mechanism, regularization, and depth-separable convolution for the YOLOX model. The results showed that the algorithm improved the average detection performance, the speed enabled real-time detection, and the performance focused on complex and small background targets. Based on YOLOv8n, Liu et al. [42] proposed a model to support the safe operation of power systems by addressing the processing and lightweight design challenges for the effective real-time detection of transmission line anomalies by drones and other resource-constrained mobile devices.

Hien et al. [43] proposed an architecture to improve the ability to recognize faulty insulators by enhancing the feature extraction network to accurately localize insulators in visually complex backgrounds. This was performed by introducing a new architecture for object detection and incorporating a diffusion model that enables context-aware inference while preserving linear scalability. Recently, an improved tilted insulator detection based on YOLOv8 was proposed by Wang et al. [44] and tested on real power grid insulators with high detection performance.

YOLOv12 has the attention-oriented mechanisms, including area attention and residual efficient layer aggregation, to improve feature representation [45]. The YOLO26 refines label assignment strategies for small object detection and introduces the multi-scale stochastic gradient descent (MuSGD) optimizer, aiming to improve deployment efficiency [21]. Applications of YOLO26 for insulator detection, as proposed in this paper, are still rare.

### Data augmentation for power system analysis

A major challenge in applying DL-based models to identify faulty insulators is that the fault classes have few samples [46]. Insulators without faults are rare to find in the network, making it difficult to train DL-based models to identify them. One of the solutions that can be employed to improve the ability to deal with unbalanced data and augmentation through generative models [47].

A dataset of 13,000 photos of synthetic foggy insulators was presented by Zhang et al. [48]. By adding the channel attention mechanism in the YOLOv5 network, a reliable detection model for insulators and their flaws is created. Using a unique fully convolutional network architecture, Sampedro et al. [49] proposed an automatic solution to diagnose and recognize insulator strings. Synthetic photos and sequences of actual aerial inspections of high-voltage insulators were used to evaluate the proposed procedure.

To segment insulators at the pixel level in real-time, a compact end-to-end neural network trained within the context of conditional GAN (cGAN) is suggested by Chang et al. [50]. Superior segmentation and real-time performance are demonstrated by the experimental results. To create segmentation samples for the insulators, including positive, empty, and fake ones, Chang et al. [51] proposed a synthetic approach. Three end-to-end segmentation networks were used by Chang et al. [50] to adjust to the generators in an adversarial training framework to validate these generated samples, which are consistent with the earlier studies.

For pixel-level insulator segmentation, a modified cGAN was proposed by Gao et al. [52]. To simplify the network complexity and extract additional types of feature information, the generator is recreated using encoder-decoder layers with an asymmetric convolution kernel. Experiments confirm that the suggested approach outperforms existing state-of-the-art networks in terms of computing efficiency.

Kim et al. [53] suggested using a deep convolutional GAN as a generative method to generate a large number of line-post insulator defect images. Chen et al. [54] employed the diffusion model for perceptual interpretation of images to improve the model’s effectiveness in insulator fault identification. The proposed model outperforms Faster R-CNN and DETRs. Li et al. [55] developed an insulator defect detection model based on improved YOLOv5s, which significantly reduces model parameters while maintaining detection performance. Additionally, the visual object classes dataset and the synthetic faulty insulator dataset were used to validate the generalization of the model.

Zhou et al. [56] collected datasets based on composite insulator surface images. Three GANs were used to adapt to the image data and synthesize virtual representations of the insulator surface. Liu and Huang [57] introduced multi-domain insulator multi-scale spatial augmentation to assist the target model in more correctly locating faults at various sizes without the need for additional inference resources, which is important for transmission lines. Yang et al. [58] proposed a high-precision technique based on the synthetic weather algorithm and enhanced YOLOv7 to detect insulator flaws in extreme weather conditions. The dataset was enhanced with synthetic rain, snow, and fog algorithms. Besides, the original dataset was augmented with affine and color adjustments to boost the generalization of the model’s performance in complex power inspection backdrops.

Jiang et al. [59] proposed a weakly-supervised learning-based automatic augmentation method for autonomously generating enhanced aerial pictures using an existing dataset and backdrop images. To ensure diversity and avoid manual costs, the weakly-supervised segmentation mix employs augmentation and segmentation techniques. Zhang et al. [60] employed an enhanced GAN to generate artificial defective insulator samples, which greatly reduces the need for substantial empirical data collection. Several experiments were run to determine the quality of the generated samples, as well as their impact on the detection model.

Ning et al. [61] expanded the glass insulator data using the enhanced denoising diffusion probabilistic models generator. The augmented noise-fitting network produces images of insulator defects with high fidelity and increased quality. Faster R-CNN was selected as the model for defect detection, and ResNet-50 was used instead of the VGG-16 feature extraction network. A DL framework that combines YOLO with deep convolutional GANs and super-resolution GANs was a promising model for the detection and classification of insulator scenarios presented by Akella et al. [62]. In comparison to the state-of-the-art techniques, a lightweight adaptive method for detecting insulator self-explosion faults was presented by Liu and Jiang [63], and it proved to be a promising alternative.

## Methodology

**Figure 1:**
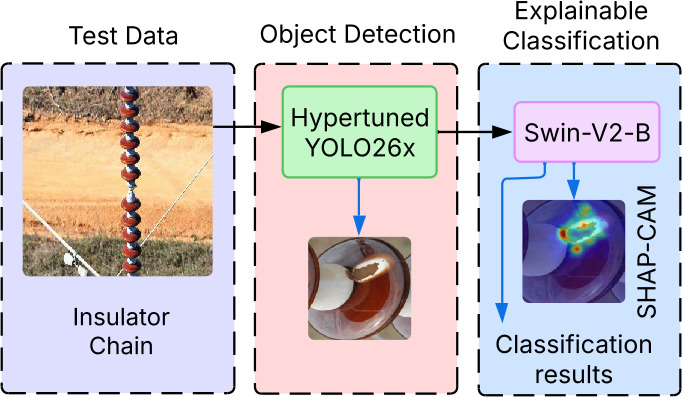
Summarized pipeline of the inference of the proposed hybrid YOLO26-Swin method

In this paper, we propose a cascaded framework that separates object detection and classification tasks. Figure 1 presents a summarized structure of the proposed model, called YOLO26-Swin, employed for inference. The proposed structure considers a hypertuned YOLOv26x to identify the insulator position, and a Swin-V2-B to perform classification. In the inference, augmented data, i.e., synthetic images, are not used; only real images recorded during the electrical inspection of the system are considered.

**Figure 2:**
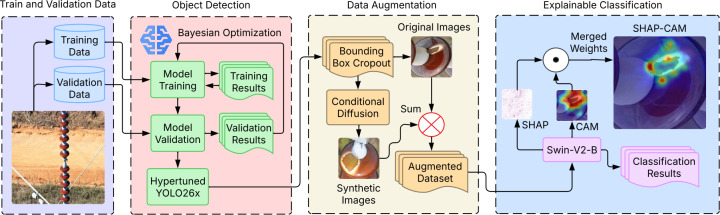
Summarized pipeline of the training of the proposed YOLO26-Swin method

During training, the YOLOv26x has its hyperparameters tuned by Bayesian optimization to ensure its best use. Considering that the dataset has unbalanced classes, synthetic images generated by conditional diffusion are considered to augment the dataset. The Swin-V2-B is used for classifying the insulator conditions, and a proposed SHAP-CAM is used to provide interpretable results. All the components of this hybrid structure are explained in this section. Figure 2 shows the summarized pipeline of our model considering the training phase.

The major highlights of our proposed model are the following:

- We proposed a YOLO26-Swin to perform identification of insulators and classification of faults separately, as explained in Sect. 3.1.
- To ensure the best use of the model setup, the Bayesian optimization is used for hyperparameter tuning, presented in Sect. 3.2.
- Considering that the dataset is unbalanced, we used the conditional diffusion model to augment the dataset, creating synthetic images of faults (see Sect. 3.3).
- To have more explainability, we proposed a SHAP-CAM, enhancing the explainability of the results; we present more details in Sect. 3.4.

### Proposed YOLO26-Swin

The proposed YOLO26-Swin considers YOLO for insulator detection. The Swin architecture is used based on the bounding boxes found in the object detection phase. Combining YOLO26 with Swin creates a modified model (YOLO26-Swin), which has separate detection and classification tasks. These methods are explained in this subsection.

#### Object detection using YOLO

DL has revolutionized object detection by enabling highly accurate and efficient recognition of objects in images, using CNN-based architectures like DETR, Faster R-CNN, RetinaNet, SSD, and YOLO. These models leverage large annotated datasets to learn features, allowing them to distinguish objects of varying sizes and orientations. Among these models, the YOLO stands out for its superior performance in object detection [64].

YOLO is a single-stage object detector that reframes object detection as a regression problem. Given an input image, YOLO divides the image into an $S \times S$ grid. Each grid cell is responsible for predicting *B* bounding boxes, their corresponding confidence scores, and the conditional class probabilities [65].

For each grid cell, the network predicts: The coordinates of the bounding box ($\hat{b} = (x, y, w, h)$, where $(x, y)$ denotes the bounding box center relative to the grid cell boundaries, while *w* and *h* correspond to the width and height (often normalized by the image dimensions). The confidence score is given by

1 $$\begin{aligned} \hat{c} = \Pr (\mathrm{object}) \cdot \mathrm{IoU}(b, \hat{b}), \end{aligned}$$

which represents both the probability that an object exists in the bounding box and the performance of the bounding box prediction as measured by the IoU between the predicted box *b̂* and the ground truth box *b*. Conditional class probabilities for each class *c* are

2 $$\begin{aligned} \hat{p}_{c} = \Pr (\text{class } c \mid \mathrm{object}). \end{aligned}$$

The final output tensor of the network has the shape $(S \times S \times \left (B \cdot 5 + C\right ))$, where factor 5 comes from the four bounding box coordinates plus one confidence score per box, and *C* is the number of classes [66]. The overall loss function in YOLO is designed to balance localization error, confidence error, and classification error.

These errors are calculated by the localization loss (Eq. (3) and Eq. (4)), confidence loss (Eq. (5)), classification loss (Eq. (6)). Localization loss measures the error in predicting the box center coordinates and dimensions, given by

3 $$L_{\text{coord}}=\lambda_{\text{coord}}\sum_{i=1}^{S^{2}}\sum_{j=1}^{B}1_{ij}^{\text{obj}}[{\left(x_{i}-{\hat{x}}_{i}\right)}^{2}+{\left(y_{i}-{\hat{y}}_{i}\right)}^{2}],$$

and for the width and height

4 $$L_{\text{size}}=\lambda_{\text{coord}}\sum_{i=1}^{S^{2}}\sum_{j=1}^{B}1_{ij}^{\text{obj}}[{\left(\sqrt{w_{i}}-\sqrt{{\hat{w}}_{i}}\right)}^{2}+{\left(\sqrt{h_{i}}-\sqrt{{\hat{h}}_{i}}\right)}^{2}],$$

where $1_{ij}^{\text{obj}}$ denotes a binary indicator that takes the value 1 if the *j*th bounding box predictor in cell *i* is assigned to detect an object, and 0 otherwise. The square root transformation helps stabilize gradients for larger boxes.

The confidence loss ($L_{\mathrm{conf}}$) penalizes the difference in the confidence score for boxes with and without objects, calculated according to

5 $$L_{\text{conf}}=\sum_{i=1}^{S^{2}}\sum_{j=1}^{B}[1_{ij}^{\text{obj}}{\left(c_{i}-{\hat{c}}_{i}\right)}^{2}+\lambda_{\text{noobj}}1_{ij}^{\text{noobj}}{\left(c_{i}-{\hat{c}}_{i}\right)}^{2}],$$

where $1_{ij}^{\text{noobj}}$ equals 1 if the *j*th bounding box in cell *i* does not have to any object.

Classification loss ($L_{\mathrm{class}}$) applies only to cells responsible for detecting objects, given by

6 $$L_{\text{class}}=\sum_{i=1}^{S^{2}}1_{i}^{\text{obj}}\sum_{c\in \text{classes}}{\left(p_{i}(c)-{\hat{p}}_{i}(c)\right)}^{2}.$$

where $\lambda _{\mathrm{coord}}$ and $\lambda _{\mathrm{noobj}}$ are hyperparameters used to balance the contributions of the localization and confidence errors [67]. Thus, the total loss is

7 $$\begin{aligned} L = L_{\mathrm{coord}} + L_{\mathrm{size}} + L_{\mathrm{conf}} + L_{ \mathrm{class}}. \end{aligned}$$

The localization loss (Eq. (3) and Eq. (4)) measures the error in the bounding box coordinate predictions (with a square root applied to *w* and *h* to lessen the impact of large errors). The confidence loss (Eq. (5)) accounts for the error in the confidence scores when an object is present or absent, respectively. The classification loss (Eq. (6)) measures the classification error only for the grid cells that are responsible for an object.

YOLO produces a tensor that encodes spatial information by means of a grid, and its loss function is a weighted sum of errors in localization, confidence, and classification [68]. This approach allows YOLO to achieve real-time performance with competitive performance. In this paper, YOLO is applied specifically for object detection, and classification is performed by specific CNNs, as explained in the following.

#### Classification using Swin

The hierarchical vision transformer using Shifted windows [69], known as Swin, builds upon the vision transformer framework by introducing a hierarchical structure that computes self-attention within local windows, significantly reducing computational complexity while preserving the ability to capture both local and global information. Its shifted window mechanism allows for cross-window connections that enable effective multi-scale feature learning.

Unlike traditional vision transformers that operate on fixed-size patches globally, the Swin introduces a shifted windowing scheme, allowing localized self-attention within non-overlapping windows while allowing connections across windows. This design scales better to high-resolution inputs and preserves spatial hierarchies through a patch-merging mechanism, making it highly effective for image classification tasks. By progressively aggregating information from local to global contexts, the Swin achieves promising performance across various benchmarks, outperforming several CNNs models [70].

### Hypertuning by Bayesian optimization

Bayesian optimization constructs a probabilistic model of the objective function and leverages it to identify promising hyperparameter combinations based on a trade-off between exploration and exploitation. By updating the model with each evaluation, it efficiently narrows down the search space and converges toward optimal settings [71]. This makes it well-suited for tuning our proposed model.

In the context of hyperparameter tuning, the goal of Bayesian optimization is to find the hyperparameters that minimize an objective function $f(\mathbf{x})$, calculated according to

8 $$ \mathbf{x}^{*} = \arg \min _{\mathbf{x}\in \mathcal{X}} f(\mathbf{x}). $$

Because each evaluation of *f* is computationally costly, Bayesian optimization builds a surrogate model to approximate *f* and intelligently selects the next hyperparameter configuration to evaluate [72]. A common choice for the surrogate model is a Gaussian process ($\mathcal{GP}$). This process places a priority over functions:

9 $$ f(\mathbf{x}) \sim \mathcal{GP}(m(\mathbf{x}), k(\mathbf{x}, \mathbf{x}')), $$

where $m(\mathbf{x})$ is the mean function, $k(\mathbf{x},\mathbf{x}')$ is a covariance kernel that measures the similarity between inputs. Given *n* observations

10 $$ \mathcal{D}_{n} = \{ (\mathbf{x}_{i}, y_{i}) \}_{i=1}^{n} \quad \mathrm{with} \quad y_{i} = f(\mathbf{x}_{i}) + \epsilon , \quad \epsilon \sim \mathcal{N}(0,\sigma _{n}^{2}), $$

where the posterior distribution at a new point **x** is Gaussian, given by

11 $$ p\big(f(\mathbf{x}) \mid \mathcal{D}_{n}, \mathbf{x}\big) = \mathcal{N}\big(\mu (\mathbf{x}), \sigma ^{2}(\mathbf{x})\big), $$

with

12 $$ \begin{aligned} &\mu (\mathbf{x}) = \mathbf{k}(\mathbf{x})^{\top }\left ( \mathbf{K} + \sigma _{n}^{2} \mathbf{I} \right )^{-1}\mathbf{y}, \\ & \sigma ^{2}( \mathbf{x}) = k(\mathbf{x},\mathbf{x}) - \mathbf{k}(\mathbf{x})^{ \top }\left ( \mathbf{K} + \sigma _{n}^{2} \mathbf{I} \right )^{-1} \mathbf{k}(\mathbf{x}), \end{aligned} $$

where

13 $$ \mathbf{k}(\mathbf{x}) = [k(\mathbf{x},\mathbf{x}_{1}),\dots ,k( \mathbf{x},\mathbf{x}_{n})]^{\top } $$

and **K** is the $n \times n$ kernel matrix with entries $K_{ij} = k(\mathbf{x}_{i}, \mathbf{x}_{j})$ [73].

The Expected Improvement (EI) guides the selection of the next hyperparameter to evaluate, defined as

14 $$ \mathrm{EI}(\mathbf{x}) = \mathbb{E}\left [ \max \left (0, f_{\min} - f( \mathbf{x})\right ) \right ], $$

where

15 $$ f_{\min} = \min _{1\le i \le n} f(\mathbf{x}_{i}) $$

is the best-observed value so far. Since the posterior $f(\mathbf{x}) \sim \mathcal{N}(\mu (\mathbf{x}),\sigma ^{2}( \mathbf{x}))$, EI can be written in closed form:

16 $$\begin{aligned} \mathrm{EI}(\mathbf{x}) &= (f_{\min} - \mu (\mathbf{x}))\,\Phi \left ( \frac{f_{\min} - \mu (\mathbf{x})}{\sigma (\mathbf{x})}\right ) \\ &\quad {} + \sigma (\mathbf{x})\,\phi \left ( \frac{f_{\min} - \mu (\mathbf{x})}{\sigma (\mathbf{x})}\right ), \end{aligned}$$

where *μ* is the predicted mean of the objective function, *σ* is predicted standard deviation (uncertainty), $\Phi (\cdot )$ and $\phi (\cdot )$ denote the cumulative distribution function and the probability density function of the standard normal distribution, respectively [74].

Algorithm 1 shows how Bayesian Optimization is computed considering a Gaussian process ($\mathcal{GP}$).

**Algorithm 1:**
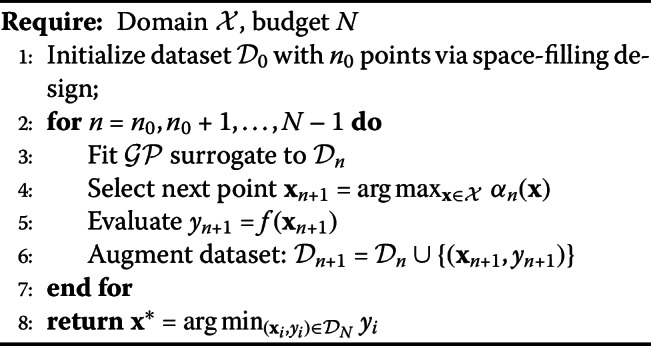
Bayesian optimization

### Data augmentation using conditional diffusion

Data augmentation using Generative Artificial Intelligence (GenAI) is an approach that enhances ML models by artificially increasing the size and diversity of training datasets [75]. GenAI models, such as GANs [76] and diffusion models, can create synthetic yet realistic data samples, including images of electrical power grid components. Data augmentation helps improve model generalization, especially in scenarios with limited data availability [77]. In this paper, a conditional diffusion model is used for data augmentation. This model is explained in the following.

Diffusion models belong to a class of generative models that generate data by learning to reverse a stochastic diffusion process. The idea is to gradually add noise to data during training and then learn to reverse this process to generate new samples [78]. The forward diffusion process adds Gaussian noise to data over *T* time steps. Given an initial data sample $x_{0} \sim q(x)$, the forward process is defined as

17 $$ q(x_{t} \lvert x_{t-1}) = \mathcal{N}(x_{t}; \sqrt{1 - \beta _{t}} x_{t-1}, \beta _{t} I), $$

where $\beta _{t}$ is a variance schedule controlling the amount of noise added at each step. Using the reparameterization, the noisy sample at any timestep *t* can be directly computed as

18 $$ x_{t} = \sqrt{\bar{\alpha}_{t}} x_{0} + \sqrt{1 - \bar{\alpha}_{t}} \epsilon , $$

where $\epsilon \sim \mathcal{N}(0, I)$ and $\bar{\alpha}_{t} = \prod _{s=1}^{t} (1 - \beta _{s})$.

The goal of the model is to learn the reverse process:

19 $$ p_{\theta}(x_{t-1} \lvert x_{t}) = \mathcal{N}(x_{t-1}; \mu _{\theta}(x_{t}, t), \Sigma _{\theta}(x_{t}, t)). $$

Diffusion models provide a framework for generative modeling by learning to reverse a noise-adding process [79]. This paper considers a conditional diffusion model; thus, the model generates images of synthetic insulators of the flashover and broken classes in a single run. The focus is on generating images of faults because the number of faults is much smaller than that of insulators in good condition, so it is possible to have a more balanced dataset.

### Explainability using the proposed SHAP-CAM

In this study, SHAP is merged with CAM, named SHAP-CAM, enabling a more comprehensive and interpretable analysis of model behavior, particularly in complex tasks such as image classification or object detection. SHAP provides global feature importance, while CAM provides spatial insights. Together, they provide both feature-level and region-level explanations. SHAP-CAM is used to highlight the regions of an input image that are most influential to the model’s decision [80].

#### SHAP

To improve the interpretability and transparency of DL models, the SHapley Additive exPlanations approach [81], in short SHAP, is applied. SHAP is based on the concept of Shapley values from cooperative game theory, which attribute the contribution of each player (feature) to the total payoff (model output) by considering all possible feature combinations.

Shapley values measure the individual contribution of each participant within a cooperative system. Variables to classify insulator defects are considered as participants within the SHAP structure. When a new variable is introduced into the model, SHAP calculates its marginal contribution by considering how it influences the outcome across various permutations of the variable order [82]. This process allows for a comprehensive assessment of the significance of each variable, facilitates the analysis of individual responses to changes in influencing factors, and provides insight into how variable interactions influence the final prediction.

Given a model *f* and an input instance *x*, SHAP computes the contribution $\phi _{i}$ of each feature *i* by averaging the marginal contributions of that feature over all possible subsets of features. Formally, the Shapley value for feature *i* is defined as

20 $$ \phi _{i} = \sum _{S \subseteq F \setminus \{i\}} \frac{\lvert S\lvert ! (\lvert F\lvert - \lvert S\lvert - 1)!}{\lvert F\lvert !} \left [ f(S \cup \{i\}) - f(S) \right ], $$

where *F* is the set of all features, *S* is a subset of features excluding *i*, and $f(S)$ represents the model’s output when only the features in *S* are considered [83].

#### CAM

CAM methods [84] allow the visualization of the spatial regions in the input that contribute most to the model’s predictions. These methods generate saliency maps that highlight relevant features, assisting domain experts in validating the model’s focus areas and ensuring that the decisions are based on meaningful patterns rather than artifacts or noise. In this paper, a set of advanced CAM techniques was applied to understand the outputs of our proposed YOLO26-Swin model. The CAM methods evaluated include eXtended Gradient-weighted CAM (XGradCAM), High-Resolution CAM (HiResCAM), and Score-weighted CAM (ScoreCAM).

XGradCAM extends the original Grad-CAM [85] formulation by introducing a more stable normalization of the gradient-based weights. Instead of relying solely on the average gradients, XGradCAM applies an alternative normalization scheme to mitigate the impact of vanishing or unstable gradients. Although XGradCAM lacks a formal peer-reviewed publication, it has been incorporated into practical implementations such as the pytorch-grad-cam library, where it demonstrates improved stability and consistency over Grad-CAM.

HiResCAM [86] proposes a gradient-free approach that utilizes the raw activations of the convolutional layers to produce high-resolution saliency maps. By bypassing the need for backpropagation, HiResCAM reduces computational overhead and enables finer spatial localization. However, since it does not incorporate gradient information, it may sacrifice some fidelity in representing the true contribution of the highlighted regions to the model’s output.

ScoreCAM, introduced by Wang et al. [87], eliminates the reliance on gradients by using the model’s output scores to weight the importance of each activation map. This is achieved by masking the input image with individual activation maps and measuring the change in the model’s output score for the target class. While ScoreCAM provides high-resolution and robust saliency maps, it is computationally expensive as it requires multiple forward passes through the network for each activation map.

#### Combining SHAP with CAM

Algorithm 2 shows the hybrid visual explanation technique that combines SHAP values with CAM (our proposed SHAP-CAM) to improve the interpretability of deep neural network predictions. Initially, the input image is transformed and passed through a trained model to obtain the predicted class. Using this class as a target, a CAM heatmap is generated via CAM methods (XGradCAM, HiResCAM, or ScoreCAM).

**Algorithm 2:**
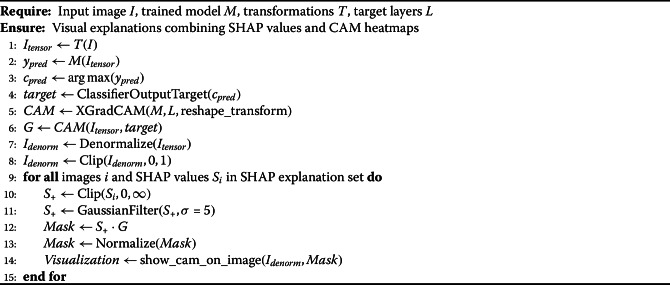
SHAP-CAM visual explanation generation

SHAP values are computed and filtered to retain only positive contributions, which are then smoothed. The final explanation is produced by element-wise multiplication of the CAM and SHAP maps, followed by normalization and overlay on the original image, yielding a combined visualization that highlights the most relevant regions both in terms of class activation and feature importance.

The integration of SHAP and CAM in Algorithm 2 is performed through a complementary fusion mechanism that combines feature attribution with spatial localization. Specifically, the CAM heatmap *G* encodes class-discriminative spatial regions derived from the network activations, while the SHAP values $S_{i}$ quantify the contribution of input features to the model output.

To ensure interpretability, only the positive SHAP contributions are retained and smoothed via a Gaussian filter to reduce noise. The fusion is then carried out through an element-wise multiplication between the processed SHAP map $S_{+}$ and the CAM heatmap *G*, i.e., $\mathrm{Mask} = S_{+} \odot G$, which emphasizes regions that are simultaneously important in both attribution spaces.

This operation acts as a gating mechanism, preserving only the spatial locations that are supported by both feature importance and activation strength. Finally, the resulting map is normalized to the $[0,1]$ range to ensure consistent visualization and comparability across samples, and overlaid on the original image. This formulation improves robustness and reproducibility by explicitly defining how SHAP and CAM are jointly leveraged to produce coherent visual explanations.

## Experimental setup

The experiments were performed using an NVIDIA RTX 3060 TI graphics processing unit with 120 GB of random-access memory. The algorithms proposed here were written using the Python language. For object detection, a maximum of 500 training epochs was defined, using the early stop criterion when there is no improvement in training after 100 epochs.

For the generation of synthetic images using generative models, a maximum of 5000 epochs was considered, and the performance was evaluated according to the number of epochs for training to define how many epochs are needed to train the model to achieve the best performance. For future comparisons, the proposed algorithm is available at: https://github.com/jpmcarvalho/Optimized-YOLO.

### Performance evaluation metrics

For model assessment, the results of precision (21), recall (22), F1-score (23), and mean average precision (mAP) (24), are considered. Specifically, the IoU threshold applied for object detection is 0.5 (mAP@[0.5]) and from 0.5 to 0.95 (mAP@[0.5:0.95]), achieved by calculating the average of the results using these thresholds with a step of 0.05. These metrics use the true positive (*tp*), false positive (*fp*), and false negative (*fn*), calculated according to

21 $$\begin{aligned}& \textup{precision}=\frac{tp}{tp+fp} \end{aligned}$$

22 $$\begin{aligned}& \textup{recall}=\frac{tp}{tp+fn} \end{aligned}$$

23 $$\begin{aligned}& \textup{F1-score}= \frac {2 \times \textup{recall} \times \textup{precision}}{\textup{recall}+\textup{precision}} \end{aligned}$$

24 $$\begin{aligned}& \textup{mAP}=\frac{1}{n}\sum _{k=1}^{n}\left ({\sum _{\eta}( \textup{recall}_{\eta}-\textup{recall}_{\eta -1})\textup{precision}_{ \eta}}\right )_{k} \end{aligned}$$

where *η* is the $nth$ threshold and *k* is the corresponding class of *n* classes [88].

### Models for comparison

**Figure 3:**
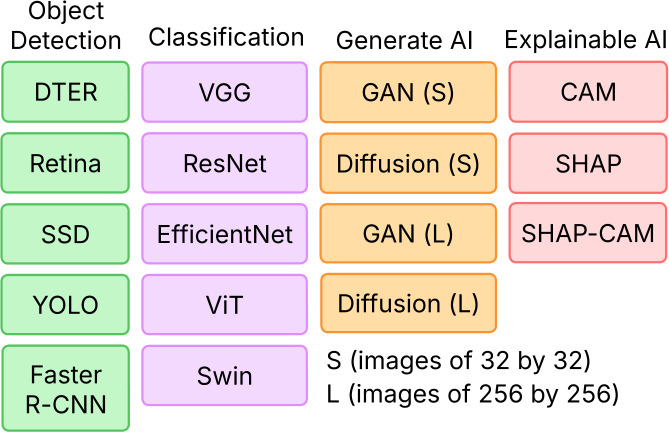
Methods compared in this paper

Figure 3 presents all the compared models evaluated in this paper. In terms of object detection, the proposed method is compared to the DETR [89], Faster R-CNN [90], RetinaNet [91], SSD [92], YOLO (10, 11, 12, and 24), STN-YOLO, and SAE-YOLO [22]. Regarding classification, the YOLO26-Swin is compared to the VGG (11, 13, 16, and 19) [93], ResNet (50, 101, and 152) [94], EfficientNet (V2-S, V2-M, and V2-L) [95], ViT (B-16, B-32, L-16, and L-32) [96], Swin (V2-T, V2-S, and V2-B).

These CNN architectures are compared based on the bounding boxes found in object detection. For data augmentation, the conditional diffusion is compared to the conditional Wasserstein GAN [97]. For explainability comparison, the SHAP, XGradCAM, HiResCAM, ScoreCAM, and SHAP-CAM (using all these CAM methods) are evaluated.

### Dataset

Images of insulators with three subclasses of condition make up the dataset: good insulator, broken insulator, and flashover insulator. These are original, high-resolution photos taken from high-voltage inspected transmission power grids. Lewis and Kulkarni’s repository [98] contains the original dataset and additional metadata for future comparisons.

**Figure 4:**
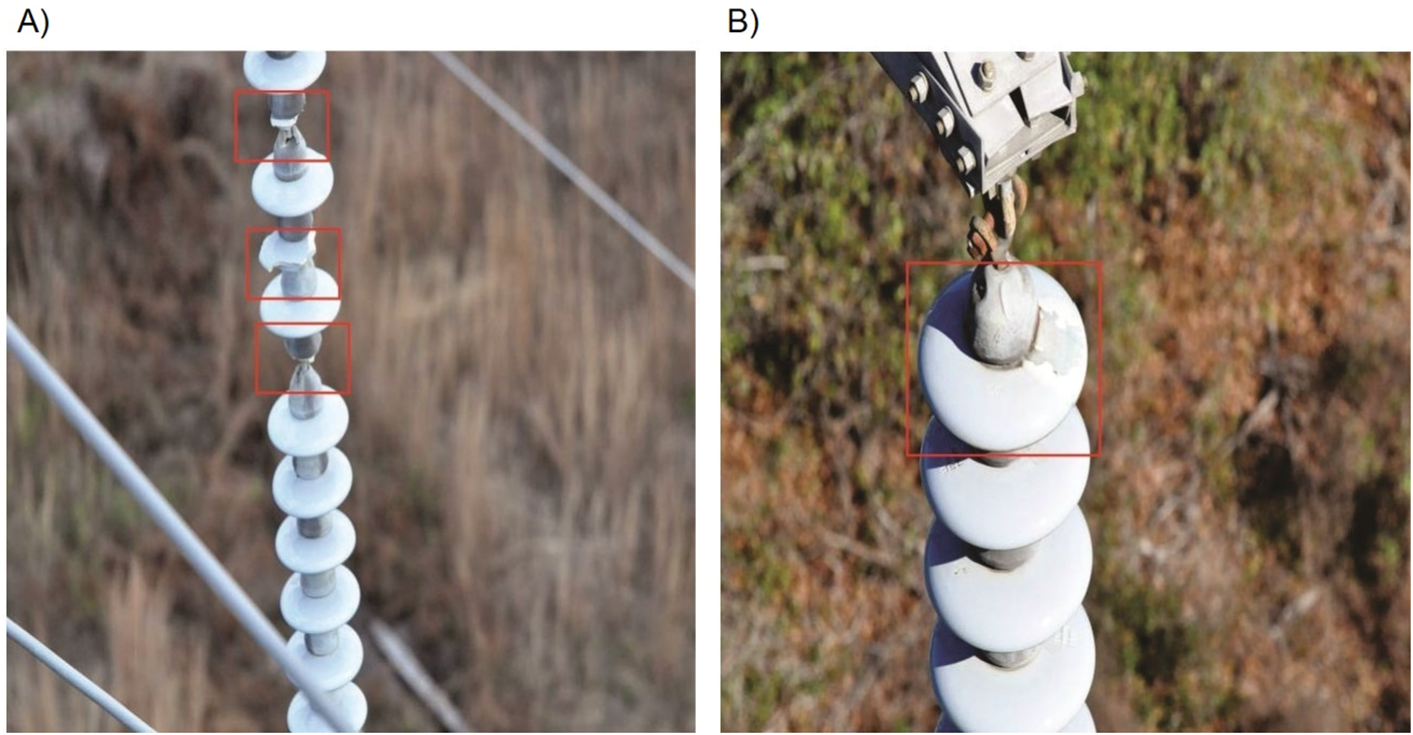
Examples of insulator fault conditions in the original dataset: (A) Broken and (B) flashover cases. Highlighted fault regions are indicated by red bounding boxes

High-resolution pictures taken by digital single-lens reflex cameras during power grid inspections in favorable weather circumstances make up the dataset under consideration. The pre-processing involved rescaling the photos to 640×640 pixels, which is the usual size for the models under consideration, and converting the annotations from JSON files to YOLO-compatible readable files (.txt). An example of the considered insulators (highlighting the damaged ones in a red box) is shown in Fig. 4.

The dataset contains 1,596 images and 17,108 annotations, with a consistent density of about 10.7 annotations per image across training, validation, and test splits. The partitioning preserves similar annotation distributions, ensuring no bias in object density between splits. Table 2 summarizes the composition of the dataset used in the experimental evaluation.

**Table 2:** Composition of the insulator fault dataset used for training, validation, and testing. Annotation counts refer to individual bounding-box instances per class

Split	Images	Annotations per class			Total ann.	Avg. ann./img
		*No issues*	*Broken*	*Flashover*		
Train	1,117	9,266	818	1,850	11,934	10.68
Validation	159	1,457	109	235	1,801	11.33
Test	320	2,641	253	479	3,373	10.54
**Total**	**1,596**	**13,364**	**1,180**	**2,564**	**17,108**	**10.72**
*Share (%)*	—	78.1%	6.9%	15.0%	100%	—

The dataset contains 1,596 images and 17,108 annotated bounding-box instances, divided into training, validation, and test subsets with 1117, 159, and 320 images, respectively. The average number of annotations per image remains consistent across the three subsets, ranging from 10.54 to 11.33, indicating a similar object density among the splits. However, the class distribution is highly imbalanced: the *No issues* class represents 78.1% of all annotations, while the *Broken* and *Flashover* fault classes account for only 6.9% and 15.0%, respectively. This imbalance highlights the difficulty of learning representative fault patterns, particularly for broken insulators, and motivates the use of synthetic data augmentation to increase the diversity and availability of minority-class samples during training.

## Results and discussion

This section presents the results and discusses the application of the proposed YOLO26-Swin. Initially, the results are evaluated in relation to the object detection task, then the classifiers are analyzed. Considering the best classifier, synthetic images are used in the training with the focus on mitigating the problem related to unbalanced classes.

Using the best classifier with the augmented dataset, interpretable results are presented with the SHAP, CAM, and the proposed SHAP-CAM to better understand the reasons why the model performed the classification. Finally, benchmarking is carried out to prove that the proposed model is adequate and it outperforms state-of-the-art DL models.

### Object detections results

The proposed YOLO26-Swin uses YOLO26 as an object detection model, focusing on identifying the position of the insulators. Here, we present the results of a comparison of YOLO, considering several versions and other DL models applied specifically for object detection. After this analysis, we present the YOLO hypertuning results.

#### Object detection model selection

Table 3 compares the insulator detection performance, in which all the conditions of the insulators are of the same class. In this initial experiment, the models are trained for up to 500 epochs using an early stop criteria in which training is finished when there is no improvement in the model after 100 epochs. For comparison purposes, a batch size equal to 4 (a higher batch size restricts the use of available hardware, see Sect. 4) was considered using the default model hyperparameters of each structure. The performance is also evaluated by Giga FLoating-point Operations Per second (GFLOPs).

**Table 3:** Insulator detection evaluation by standard models

Model	mAP		GFLOPs	Training time (h)	Inference time (ms)
	[0.5]	[0.5:0.95]			
DETR	0.90043	0.64615	234.96	1.85	15.21
Faster R-CNN	0.90212	0.64748	272.14	2.68	80.23
RetinaNet	0.90057	0.62167	245.77	2.98	65.67
SSD	0.83203	0.46371	91.28	1.15	29.26
STN-YOLO	0.00300	0.00074	10.70	3.18	9.15
SAE-YOLO	0.96098	0.80886	9.49	11.53	10.17
YOLOv10n	0.97937	0.88372	8.39	2.26	**3.31**
YOLOv10s	0.97657	0.89232	24.77	2.76	4.90
YOLOv10m	0.98193	0.90178	63.97	4.32	6.48
YOLOv10l	0.98107	0.91392	127.20	6.15	10.66
YOLOv10x	0.98236	0.91555	171.00	8.62	15.43
YOLOv11n	0.98445	0.89489	6.44	1.76	3.44
YOLOv11s	0.98194	0.89539	21.55	2.15	4.09
YOLOv11m	0.98602	0.90075	68.19	3.93	7.22
YOLOv11l	0.98348	0.89773	87.27	5.01	7.81
YOLOv11x	0.98344	0.90355	195.45	8.32	14.70
YOLOv12n	0.98273	**0.91799**	6.48	**0.77**	4.11
YOLOv12s	0.98651	0.90205	21.52	3.03	5.87
YOLOv12m	0.98202	0.89150	67.74	4.78	8.13
YOLOv12l	0.98137	0.89529	89.41	7.83	12.59
YOLOv12x	0.98311	0.89626	199.82	12.48	21.04
YOLOv26n	0.98486	0.91759	**5.77**	3.44	4.13
YOLOv26s	0.98201	0.91653	22.50	5.61	4.93
YOLOv26m	0.97665	0.89507	74.72	7.24	6.85
YOLOv26l	0.98667	0.90058	93.12	8.95	8.04
YOLOv26x	**0.98821**	0.90544	208.51	12.15	14.08

Best results in bold

The baseline models, including DETR, Faster R-CNN, RetinaNet, and SSD, present moderate detection performance. Their mAP@[0.5] values are approximately 0.90 for DETR, Faster R-CNN, and RetinaNet, while SSD exhibits a lower value of 0.83203. When considering the more restrictive mAP@[0.5:0.95], all baseline models show a significant reduction, with values ranging from 0.46371 for SSD to approximately 0.64748 for Faster R-CNN. These results indicate limited localization precision under stricter IoU thresholds. In addition, these models present relatively high computational costs, with GFLOPs exceeding 200 for most architectures and inference times reaching up to 80.23 ms for Faster R-CNN, which restricts their applicability in real-time scenarios. SAE-YOLO showed promising results, performing better than all models except the original YOLO models. STN-YOLO did not perform well in this analysis.

The YOLOv10 family introduces a substantial improvement in both detection performance and efficiency. All variants significantly outperform the baseline models, with mAP@[0.5] values exceeding 0.97 and mAP@[0.5:0.95] values above 0.88. The progression from YOLOv10n to YOLOv10x shows a consistent increase in performance, reaching 0.98236 and 0.91555, respectively. This improvement is associated with increased computational complexity, as GFLOPs rise from 8.39 to 171.00 and inference time increases from 3.31 ms to 15.43 ms. Nevertheless, even the largest YOLOv10 model remains faster than traditional detectors while achieving higher performance.

The YOLOv11 models further refine this trade-off. The smallest variant, YOLOv11n, achieves a mAP@[0.5] of 0.98445 with only 6.44 GFLOPs, representing the lowest computational cost among all evaluated models. The medium and large variants improve performance, with YOLOv11m reaching 0.98602 and YOLOv11x achieving 0.90355 in mAP@[0.5:0.95]. The inference times remain within a practical range, below 15 ms, confirming the suitability of this family for real-time applications.

The YOLOv12 family demonstrates additional improvements in localization performance. The YOLOv12n model achieves the highest mAP@[0.5:0.95] value of 0.91799 among all models with a relatively low computational cost of 6.48 GFLOPs and the shortest training time of 0.77 hours. Larger variants such as YOLOv12s reach a mAP@[0.5] of 0.98651, although at the expense of increased training and inference times. This indicates that YOLOv12 effectively enhances bounding box precision while maintaining computational efficiency in its smaller configurations.

The YOLOv26 models achieve the highest overall detection performance. In particular, YOLOv26x reaches the best mAP@[0.5] value of 0.98821, while maintaining a competitive mAP@[0.5:0.95] of 0.90544. The YOLOv26n variant presents a favorable balance, with mAP@[0.5] of 0.98486 and mAP@[0.5:0.95] of 0.91759, combined with low GFLOPs of 5.77. However, larger variants such as YOLOv26x require significantly higher computational resources, exceeding 200 GFLOPs, which approaches the complexity of traditional detectors.

From a global perspective, the results indicate that YOLO based architectures consistently outperform traditional detection models in both performance and efficiency. For maximum detection performance, YOLOv26x offers the highest mAP@[0.5]. In the structure definition phase, we considered this model as the most promising to be hypertuned, which is carried out using Bayesian optimization.

#### Hypertuning YOLO26

**Figure 5:**
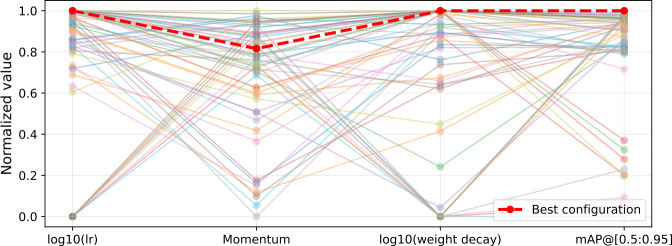
Combination of hyperparameters chosen in the model trials

Figure 5 presents the results of the multi-objective optimization trials to tune the model hyperparameters. A trial consists of choosing a value for each hyperparameter, and the lines in this plot show different combinations of values. The darker color of the lines represents the trial that performed best in terms of achieving the objective function, which in this case is the optimization of the mAP value (presented on the right side of Fig. 5).

Bayesian optimization runs 50 trials, combining the hyperparameters to achieve the model’s best performance (mAP). In this analysis, the evaluated hyperparameters were learning rate [1.0×10^−5^:1.0×10^−1^], momentum [1.0×10^−9^:0.99], optimizer [Stochastic Gradient Descent (SGD), Adaptive moment estimation (Adam), Root Mean Square propagation (RMSprop), Adam with Weight decay (AdamW), and weight decay [1.0×10^−9^:2.0×10^−1^].

**Figure 6:**
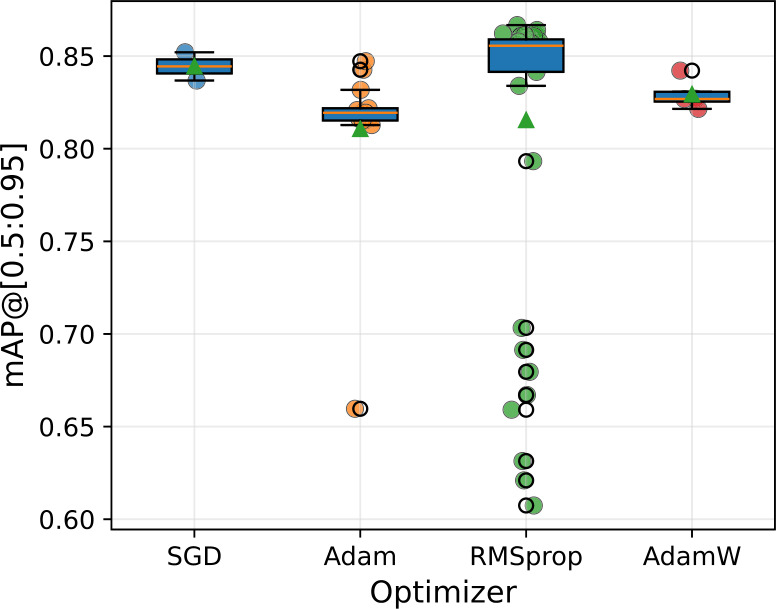
Optimizer comparison in the model hypertuning experiment

Based on this optimization experiment, the set of hyperparameters is learning rate equal to 0.01, momentum equal to 0.001, SGD optimizer, and weight decay equal to 0.0359. Using these hyperparameters, the model is trained once more for its best application. The parameter that resulted in greater variability was the choice of the optimizer, shown in Fig. 6.

RMSprop achieves the highest peak values among all optimizers, reaching around 0.86-0.87. However, this comes at the cost of substantial variability. The distribution shows a wide spread, with numerous low-performing outliers extending down to approximately 0.60. Considering that the maximum performance is prioritized and variability can be managed through extensive tuning, RMSprop is used.

**Figure 7:**
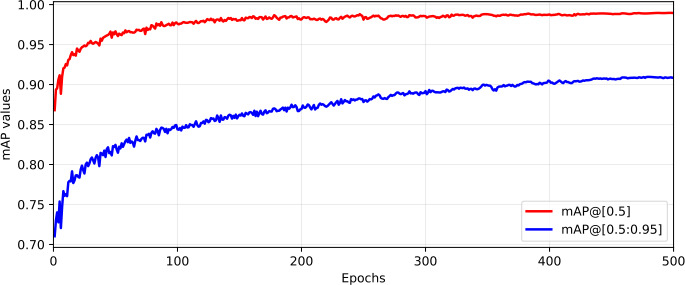
Comparison of mAP metrics over training epochs

Figure 7 shows the values of the mAP@[0.5] and mAP@[0.5:0.95] for insulator detection. It is possible to notice that it takes several epochs for the model to stabilize (after 450), highlighting how a model needs to be trained properly (until stabilized) to achieve its best performance.

### Classification results

We present the classification results for defining the base classification model. A perceived challenge in the classification phase is the low number of samples that represent faults, considering that most of the insulators are in good condition. To mitigate this problem, the conditional Wasserstein GAN and the conditional diffusion model were compared to augment the dataset, creating synthetic images of broken and flashover insulators.

#### Selection of baseline classifier

After using the optimized model to detect the insulators, the classification of faults in relation to the bounding boxes and those found is carried out. Table 4 shows the comparative results of several CNN models to select the most suitable model to be used in the classification phase. The F1-score is the main measure considered here to evaluate the classification performance.

**Table 4:** Selection of the CNN to be used for classification

Model	Precision	Recall	F1-score	GFLOPs	Training time (h)	Inference time (ms)
VGG-11	0.94597	0.90518	0.92457	128.68	2.68	**0.52**
VGG-13	0.94374	0.91627	0.92939	180.87	3.63	0.62
VGG-16	0.94444	0.91784	0.93070	247.46	4.38	0.84
VGG-19	0.92727	0.90124	0.91361	314.0	5.13	0.97
ResNet-50	0.97127	0.91866	0.94339	66.11	**2.24**	3.24
ResNet-101	0.96159	0.91532	0.93722	125.8	3.50	6.19
ResNet-152	0.96620	0.92493	0.94454	185.6	6.15	9.37
EfficientNet-V2-S	**0.98158**	0.95686	0.96886	**46.4**	2.69	9.31
EfficientNet-V2-M	0.96603	**0.97169**	0.96884	87.1	4.31	13.63
EfficientNet-V2-L	0.96601	0.94024	0.95261	198.05	5.45	22.54
ViT-B-16	0.97250	0.91986	0.94428	180.56	8.08	3.20
ViT-B-32	0.92127	0.89710	0.90872	47.2	4.80	3.09
ViT-L-16	0.95272	0.91692	0.93384	637.68	25.67	6.52
ViT-L-32	0.93065	0.90953	0.91875	163.68	14.48	6.07
Swin-V2-T	0.97511	0.94841	0.96135	47.6	4.10	8.68
Swin-V2-S	0.97584	0.95871	0.96712	92.2	6.93	16.79
Swin-V2-B	0.97692	0.96116	**0.96887**	163.58	9.90	17.51

Best results in bold

The most promising results were found using EfficientNet and the second-generation Swin. Specifically, using EfficientNet-V2-S, a precision equal to 0.98158 was achieved, and using EfficientNet-V2-S, a recall equal to 0.97169 was achieved, these being the best performance values found for the models evaluated. Considering that the F1-score takes into account both precision and recall values, this metric was chosen to determine the best model, which had a high precision and recall value simultaneously, being Swin-V2-B with an F1-score equal to 0.96887.

Observing that the images are resized to $640\times 640$ to be used in the object detection model, two analyses were evaluated for classifying insulators. The first analysis was using bounding boxes equal to $256\times 256$; in this case, considering the original high-resolution images, the second analysis was carried out considering the use of pre-processed (compressed) images, in this case with bounding boxes equal to $32\times 32$.

#### High resolution synthetic images

**Figure 8:**
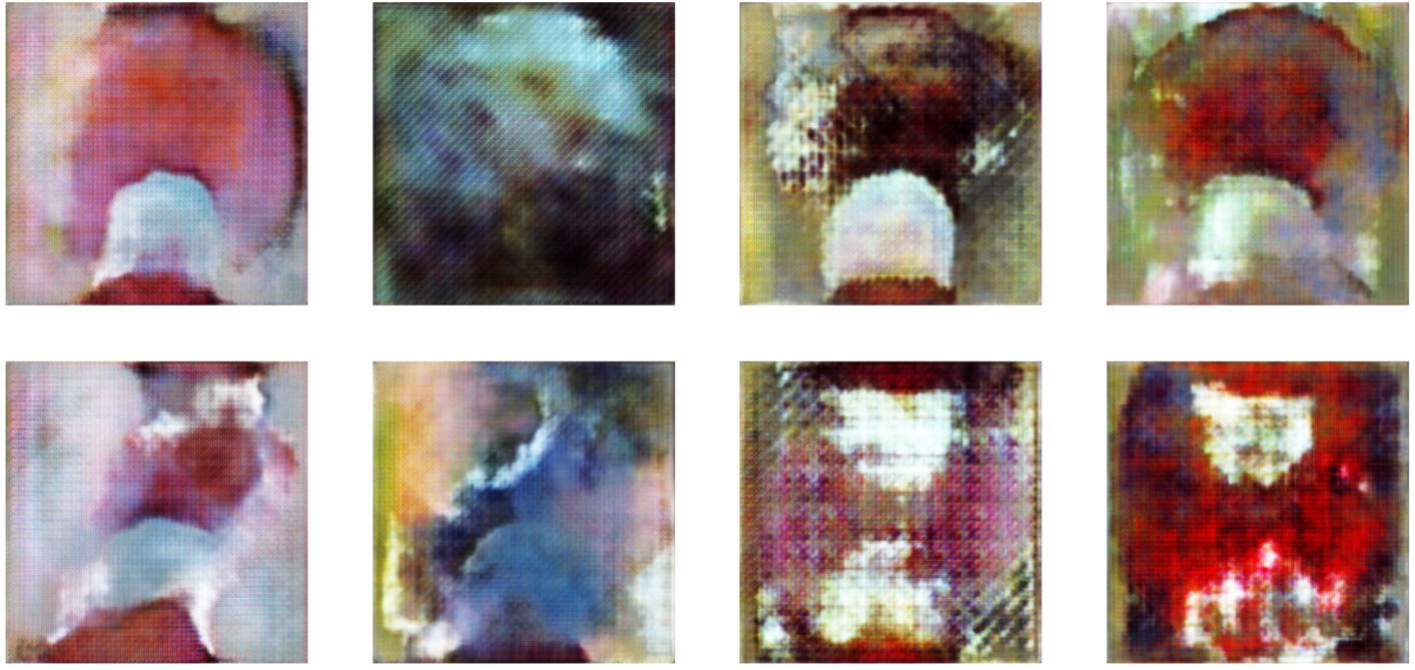
Broken insulators generated by the conditional Wasserstein GAN with a size of 256×256. The columns represent 500, 1000, 2500, and 5000 training epochs

**Figure 9:**
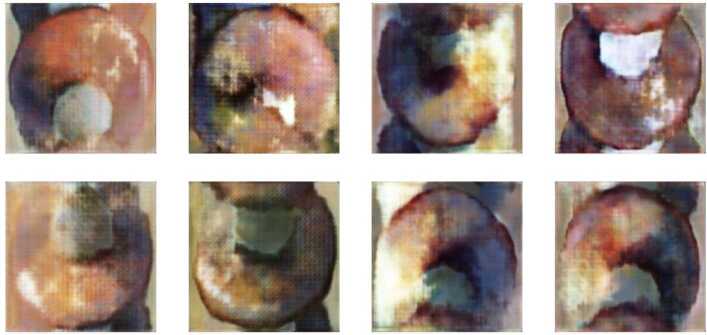
Flashover damage insulators generated by the conditional Wasserstein GAN with a size of 256×256. The columns represent 500, 1000, 2500, and 5000 training epochs

**Figure 10:**
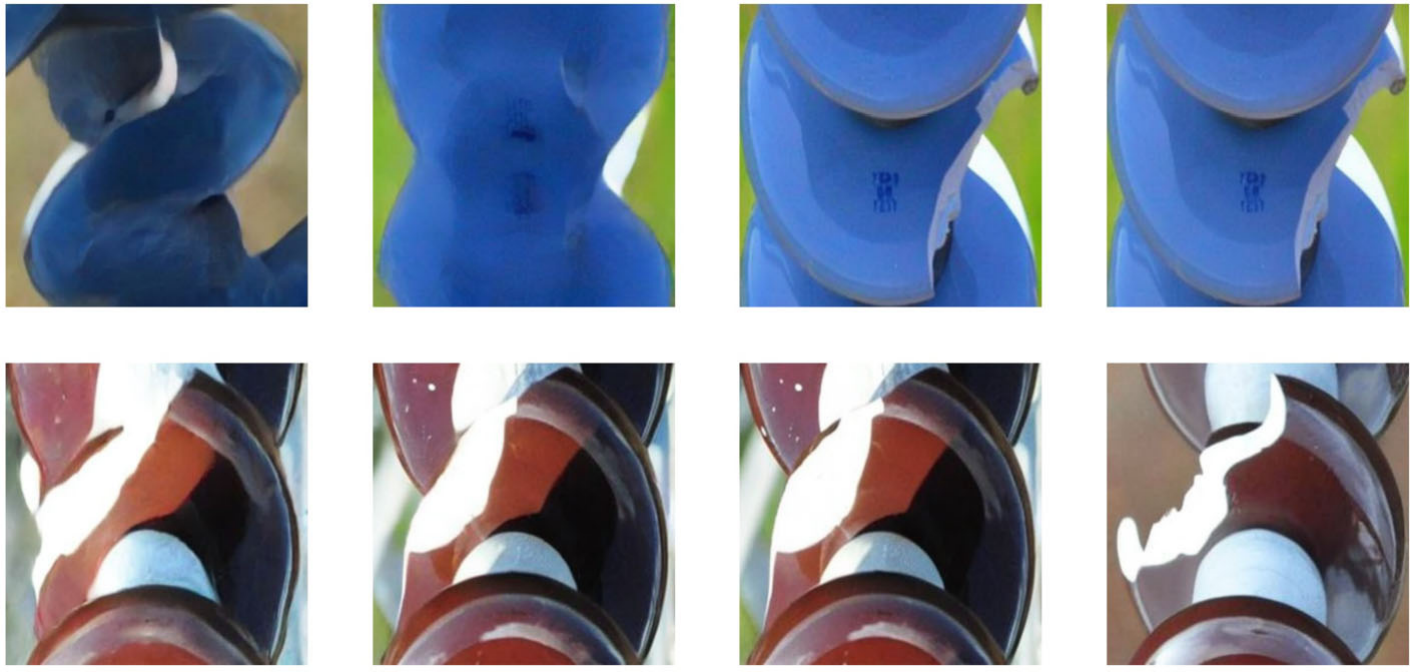
Broken insulators generated by a conditional diffusion model with a size of 256×256. The columns represent 500, 1000, 2500, and 5000 training epochs

**Figure 11:**
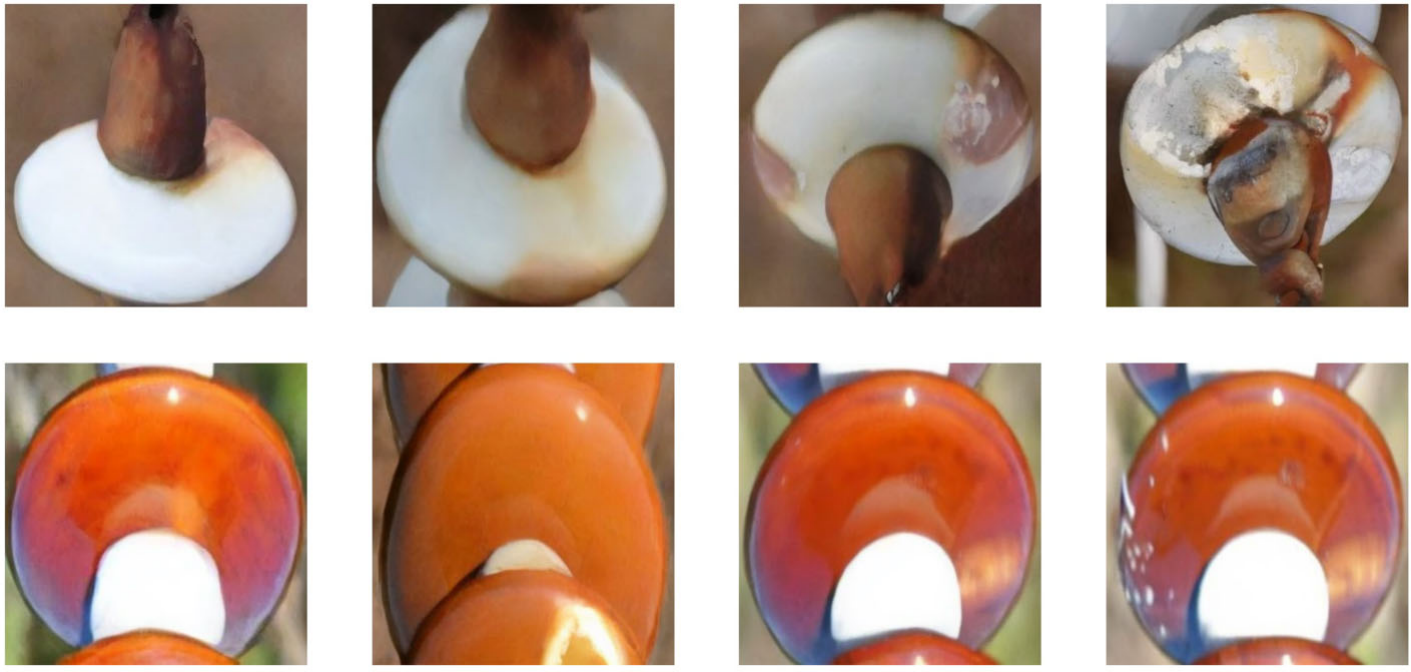
Flashover damage insulators generated by a conditional diffusion model with a size of 256×256. The columns represent 500, 1000, 2500, and 5000 training epochs

This subsection evaluates two methods of synthetic image generation, the conditional Wasserstein GAN and conditional diffusion. The synthetic images are used to augment the training dataset. The comparison considers images of $256\times 256$. Figure 8 and Fig. 9 present examples of synthetic images of $256\times 256$ generated by the conditional Wasserstein GAN, respectively of broken and flashover insulators. Comparatively, Fig. 10 and Fig. 11 present examples of images generated by conditional diffusion.

These images are presented according to the number of training epochs by each model. Qualitative results show that diffusion models are considerably superior to the conditional Wasserstein GAN. For diffusion models, it is difficult to define which images are synthetic after 2500 training epochs, which is considerably promising given that this is the aim of the augmentation carried out in this work.

Table 5 shows the quantitative results using synthetic images for the model training. This analysis considers images of $256\times 256$ pixels. The performance results are evaluated, given the number of epochs used to generate the images and the variation in the number of images used. The training time is based on the classifier model, which can vary depending on the use of the early stop.

**Table 5:** Classification results using augmented data (images of 256×256 pixels)

Model	Synthetic images	Epochs^a^	Precision	Recall	F1-score	Training time (h)
Original	-	-	0.97692	0.96116	0.96887	9.90
Conditional Wasserstein GAN	500	500	0.97972	0.97746	0.97857	24.04
		1000	0.97234	0.96703	0.96957	36.47
		2500	0.97877	0.96894	0.97376	11.74
		5000	0.98652	0.96132	0.97346	14.40
	1000	500	0.98511	0.97406	0.97949	29.41
		1000	0.98507	0.97193	0.97838	32.85
		2500	0.98117	0.97819	0.97966	20.15
		5000	0.98552	0.96759	0.97633	23.70
	2500	500	0.98343	0.97837	0.98086	35.27
		1000	0.98580	0.97500	0.98028	37.37
		2500	0.98240	0.98075	0.98157	23.28
		5000	0.98943	0.98415	0.98676	33.03
	5000	500	0.98925	0.98543	0.98731	27.24
		1000	0.98944	0.98754	0.98848	30.77
		2500	0.98939	0.98830	0.98884	21.23
		5000	0.98979	0.98928	0.98953	42.84
Conditional Diffusion	500	500	0.97354	0.98232	0.97786	20.16
		1000	0.98150	0.97883	0.98014	20.27
		2500	0.98243	0.97633	0.97933	20.25
		5000	0.96989	0.97837	0.97398	**9.72**
	1000	500	0.98339	0.98197	0.98267	22.98
		1000	0.97841	0.98554	0.98192	27.72
		2500	0.98776	0.97540	0.98147	28.09
		5000	0.99005	0.98060	0.98526	10.00
	2500	500	0.98186	0.97857	0.98017	50.10
		1000	0.98994	0.98494	0.98741	30.94
		2500	0.98647	0.97529	0.98076	22.91
		5000	0.98333	0.98841	0.98583	13.07
	5000	500	0.98855	0.98492	0.98669	45.98
		1000	0.98729	0.98515	0.98621	31.82
		2500	**0.99028**	**0.98944**	**0.98986**	18.50
		5000	0.98830	0.98837	0.98833	14.54

^a^Epochs used to create synthetic images

Best results in bold

The best result found using synthetic images was using the conditional diffusion model, trained up to 2500 epochs, using 5000 images. This result was 2.1% better than the classification model using only real images, thus justifying the use of synthetic images to train the classifier. In terms of computational effort for classification, for images of 256×256, all the models had GFLOPs equal to 163.58, considering that the same batch size (16) was used and the same classifier (Swin-V2-B). The inference time is also the same, equal to 17.51 milliseconds. The training time varied mainly due to the use of the early stop criterion.

Having the best result considering images trained up to 2500 epochs shows that training beyond this value is not necessary to achieve better results, and in this experiment, when the model was trained up to 5000 epochs, there were no major visual or numerical differences. Most of the results using the conditional diffusion model were higher than 0.98, even when a smaller number of images were used (1000 synthetic images), proving their visual and numerical superiority compared to the conditional Wasserstein GAN.

Although the images generated by the conditional Wasserstein GAN are visually different from the original images, there was still a performance improvement when these images were used. This shows that this improvement is due to the intrinsic characteristics of the classification model, since in models based on conditional Wasserstein GAN, the generator is trained to fool the classifier as to whether the images are synthetic or not.

The results of using the conditional diffusion model were promising for both broken insulators and insulators damaged by flashover. Especially for broken insulators, there was variability in the generation of different colored components with different backgrounds. This result is considerably promising since the aim of augmentation is to create a dataset that represents greater variability in the samples, so that the classification model can be used for different types of insulators.

#### Low resolution synthetic images

The purpose of using smaller images in this analysis is to compare whether the preprocessing phase impairs classification by reducing the quality of the images, noting that current YOLO models perform this operation to reduce computational complexity.

**Figure 12:**
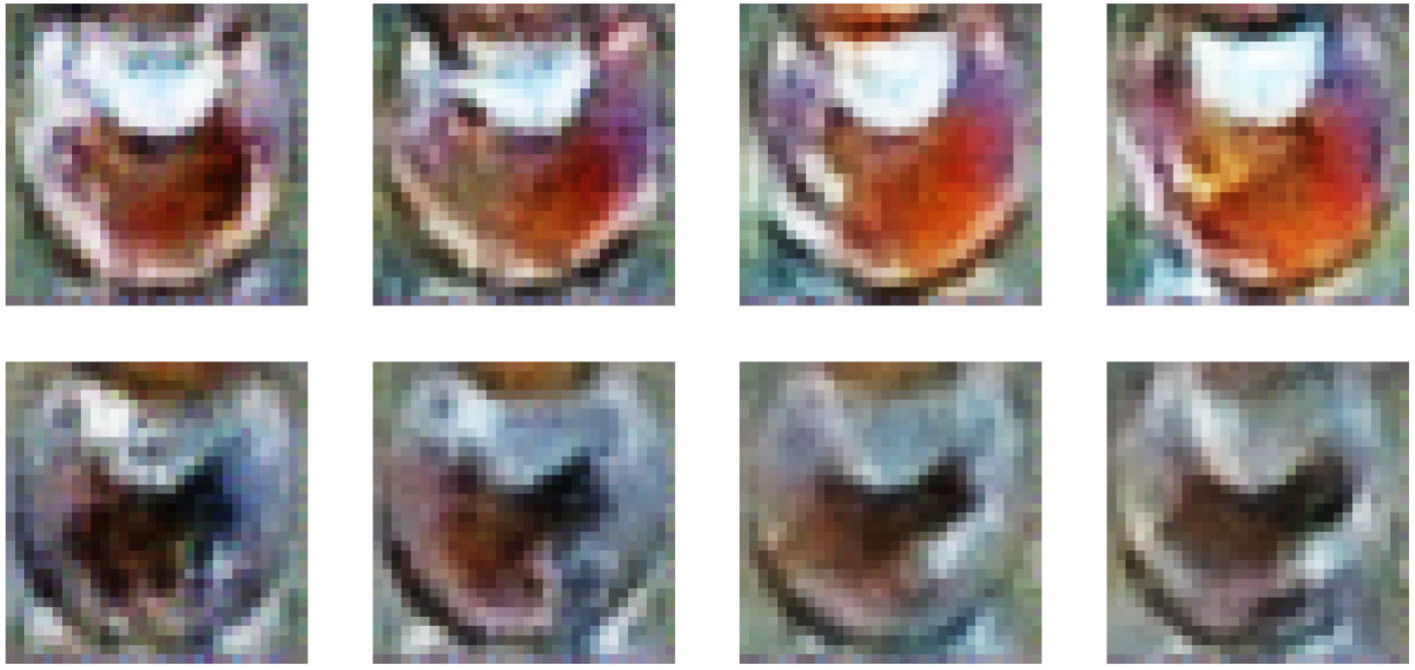
Broken insulators generated by the conditional Wasserstein GAN with a size of 32×32. The columns represent 500, 1000, 2500, and 5000 training epochs

**Figure 13:**
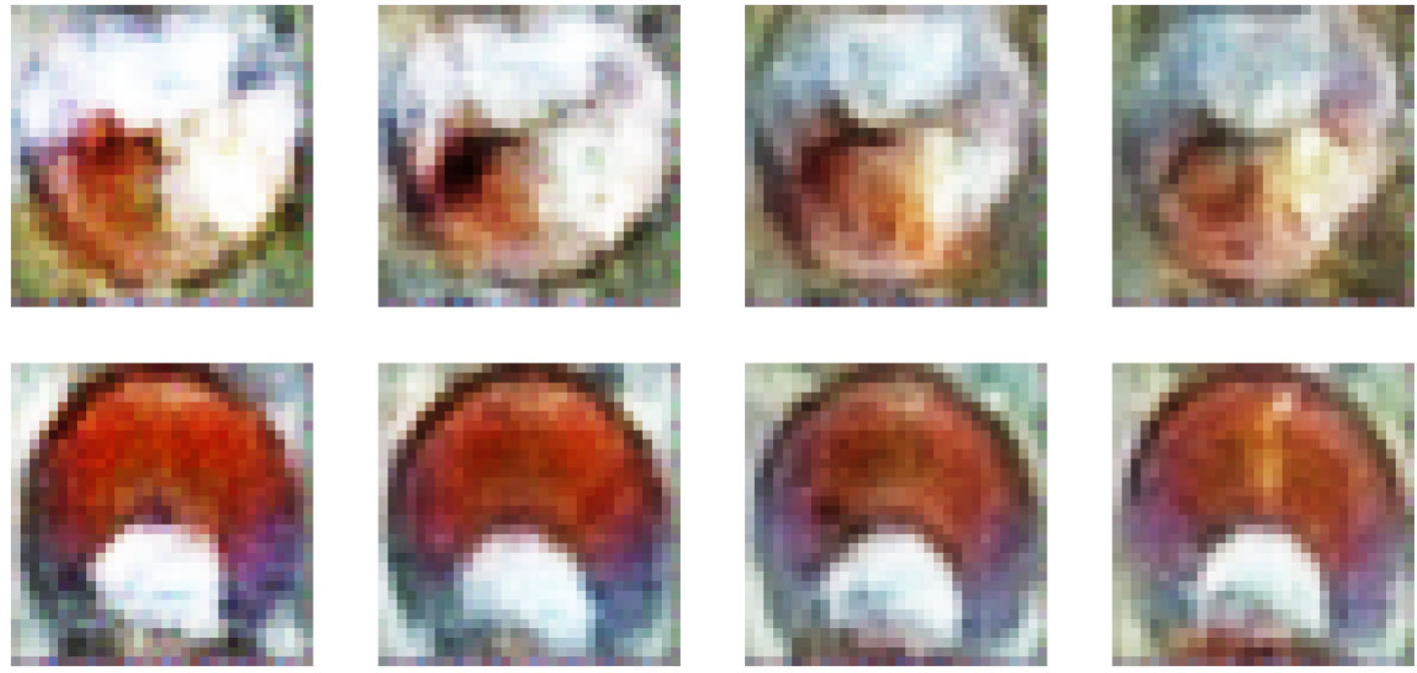
Flashover damage insulators generated by the conditional Wasserstein GAN with a size of 32×32. The columns represent 500, 1000, 2500, and 5000 training epochs

**Figure 14:**
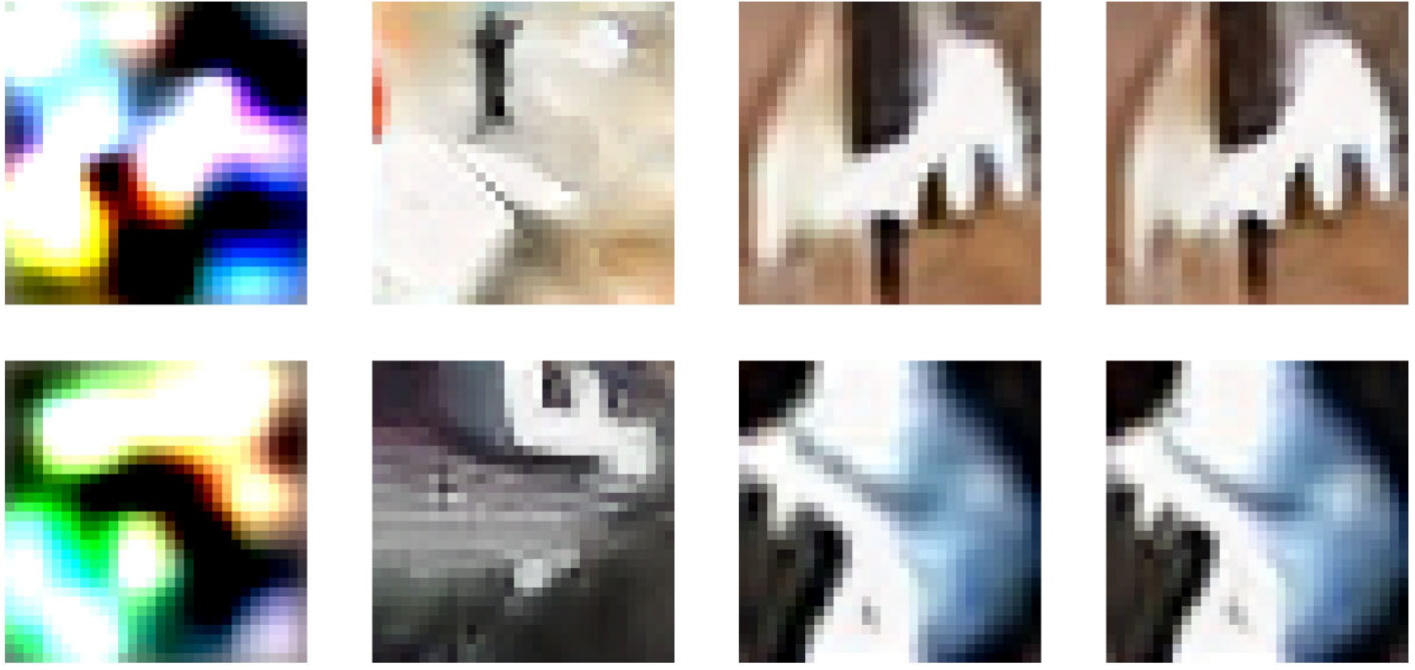
Broken insulators generated by a conditional diffusion model with a size of 32×32. The columns represent 500, 1000, 2500, and 5000 training epochs

**Figure 15:**
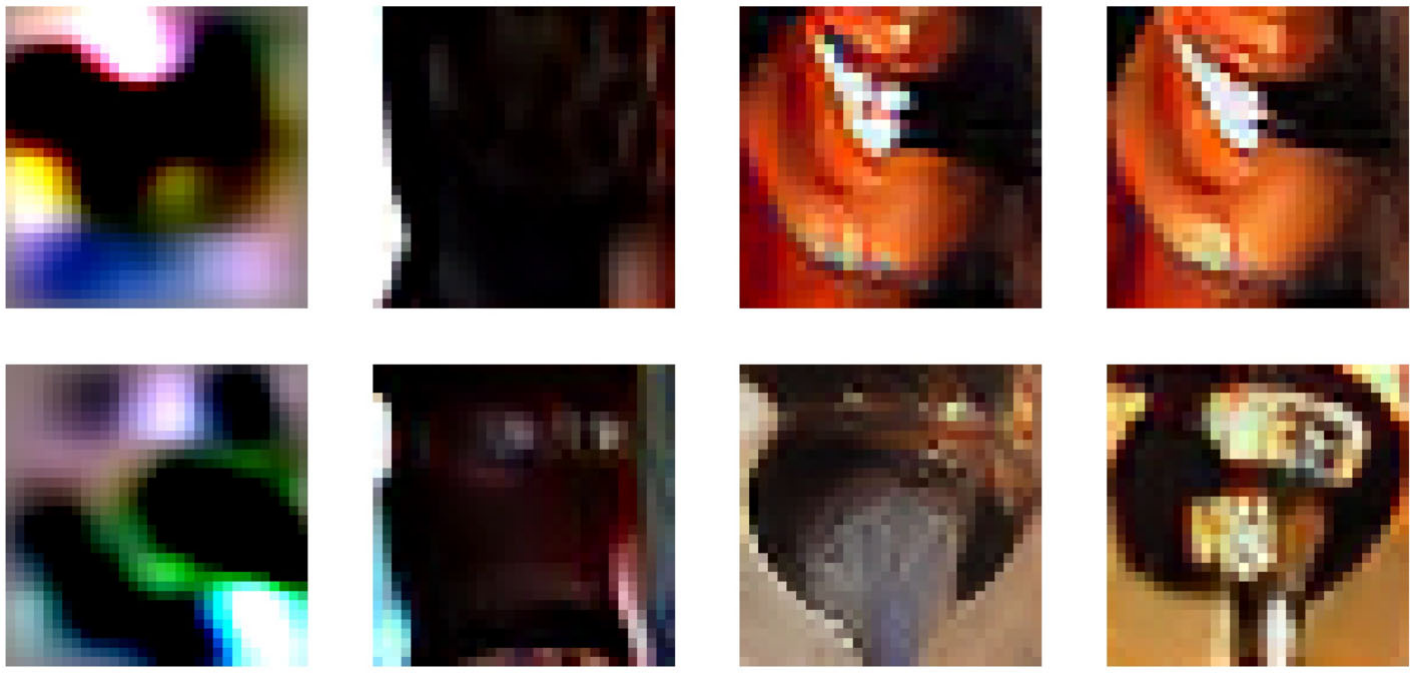
Flashover damage insulators generated by a conditional diffusion model with a size of 32×32. The columns represent 500, 1000, 2500, and 5000 training epochs

Figure 12 (broken insulators) and Fig. 13 (flashover insulators) show examples of synthetic images of $32\times 32$ generated by the conditional Wasserstein GAN. Figure 14 (broken insulators) and Fig. 15 (flashover insulators) show examples of conditional diffusion-generated images. Here, the results are also presented according to the number of training epochs by each model.

Qualitatively, the images generated by conditional Wasserstein GAN had promising results, as did the results of diffusion models after a large number of training epochs. With a low number of training epochs, the synthetic images generated by the conditional Wasserstein GAN were superior to the images generated by the conditional diffusion model, but when more than 2500 epochs are considered for generating synthetic images, the diffusion-based models show better results.

Table 6 shows the classification results using 32×32 images. The best result was found using a conditional diffusion model, achieving an F1-score of 0.98149, which is 1.26% better than the classification model (Swin-V2-B) using only original images for training. This shows that the use of synthetic images for class balancing improves the classification model’s capacity also for smaller images. In terms of computational effort, all the models had an equal GFLOPs for processing images of 32×32, considering that the same classifier (Swin-V2-B) and batch size (16) were used.

**Table 6:** Classification results using augmented data (images of 32×32 pixels)

Model	Synthetic images	Epochs^a^	Precision	Recall	F1-score	Training time (h)
Original	-	-	0.97692	0.96116	0.96887	**9.90**
Conditional Wasserstein GAN	500	500	0.95045	0.93275	0.94102	30.44
		1000	0.96351	0.93951	0.95112	36.25
		2500	0.97064	0.93759	0.95343	34.03
		5000	0.96063	0.93014	0.94476	40.34
	1000	500	0.95930	0.94886	0.95395	21.55
		1000	0.96443	0.94472	0.95415	32.47
		2500	0.96140	0.94940	0.95529	31.58
		5000	0.96751	0.95005	0.95853	42.25
	2500	500	0.96461	0.94856	0.95628	20.97
		1000	0.97813	0.95975	0.96856	36.90
		2500	0.97643	0.96523	0.97070	30.82
		5000	0.98064	0.96197	0.97096	32.48
	5000	500	0.98182	0.97309	0.97727	15.56
		1000	0.97834	0.97306	0.97562	30.95
		2500	0.98105	**0.97899**	0.97999	37.59
		5000	0.97907	0.97203	0.97537	18.94
Conditional Diffusion	500	500	0.91446	0.91227	0.91297	10.13
		1000	0.97684	0.95210	0.96409	20.43
		2500	0.96817	0.95069	0.95921	20.48
		5000	0.96259	0.93374	0.94762	10.07
	1000	500	0.92285	0.90712	0.91390	11.17
		1000	0.96938	0.95186	0.96040	32.71
		2500	0.96758	0.95010	0.95861	32.89
		5000	0.96613	0.95480	0.96037	10.11
	2500	500	0.92375	0.91887	0.92125	26.77
		1000	0.97690	0.96959	0.97319	37.18
		2500	0.97647	0.96975	0.97306	37.26
		5000	0.96604	0.96510	0.96556	23.28
	5000	500	0.93681	0.93332	0.93441	36.72
		1000	**0.98531**	0.97797	**0.98149**	44.88
		2500	0.98036	0.97496	0.97758	44.91
		5000	0.98114	0.97606	0.97853	42.02

^a^Epochs used to create synthetic images

Best results in bold

An interesting result observed using 32×32 synthetic images is that when 500 epochs are used to train the generative model, the classification results are inferior to using the original images in most cases. This shows that when a model is not trained properly, it can impair the ability to classify insulators. The only superior result using 500 training epochs to generate images was when 5000 images from the conditional Wasserstein GAN were considered, and in this case, the large number of images meant that the model’s performance was superior. The qualitative results also had this outcome, observing that when few epochs are considered, synthetic images can have worse quality. This result reinforces the importance of proper training.

In general, the use of smaller images resulted in lower performance (best F1-score equal to 0.98149) when compared to higher resolution images (best F1-score equal to 0.98986). In terms of training time, the conditional diffusion model for 256×256 images took 607.19 hours (≈ 25 days) to complete 5000 training epochs, and for 32×32 images it took 36.91 hours. In comparison, the conditional Wasserstein GAN took 120 hours (≈ 5 days) to complete 5000 training epochs, while for 32×32 images it took 22.15 hours.

A cost-benefit analysis shows that the conditional diffusion model is advantageous only when the target is maximum classification performance and the augmentation process is performed offline, whereas conditional Wasserstein GAN offers a more economical alternative when computational budget is tighter. For high resolution 256×256 images, the baseline classifier trained only on real data achieved an F1-score of 0.96887 in 9.90 h, while the best conditional Wasserstein GAN setting increased the F1-score to 0.98953 with 5000 synthetic images generated at 5000 epochs, requiring 42.84 h of classifier training; the best conditional diffusion setting further improved the F1-score to 0.98986 with 5000 synthetic images generated at 2500 epochs, while reducing classifier training time to 18.50 h.

Diffusion provided only a marginal absolute gain of 0.00033 in F1 over the best Wasserstein GAN result, but reached that best point with substantially lower downstream training time for the classifier. For low-resolution 32×32 images, however, the advantage of diffusion becomes clearer in effectiveness but not in generation cost: the original model obtained an F1-score of 0.96887, the best Wasserstein GAN result reached 0.97999, and the best diffusion result reached 0.98149, corresponding to gains of about 1.11 and 1.26 percentage points, respectively, over the non-augmented baseline. At the same time, the manuscript explicitly states that training the conditional diffusion generator required 5.06 times longer than the conditional Wasserstein GAN for 256×256 images and 16.45 times longer for 32×32 images, which confirms that the generative stage is the main computational bottleneck.

The practical conclusion is that conditional diffusion is justified when the objective is the highest possible predictive performance and augmentation is performed once offline, while conditional Wasserstein GAN remains a more favorable trade-off for adaptive or frequently retrained pipelines where lower augmentation cost is more important than the final fraction of a percentage point in F1-score.

#### Explainable results

While DL architectures such as CNNs and transformer-based models like Swin models have achieved promising performance, their decision-making processes often remain a black box. This lack of interpretability raises concerns about the reliability and trustworthiness of these models, particularly in high-stakes domains such as power grid infrastructure monitoring, where incorrect decisions can lead to costly operational failures or safety hazards.

**Figure 16:**
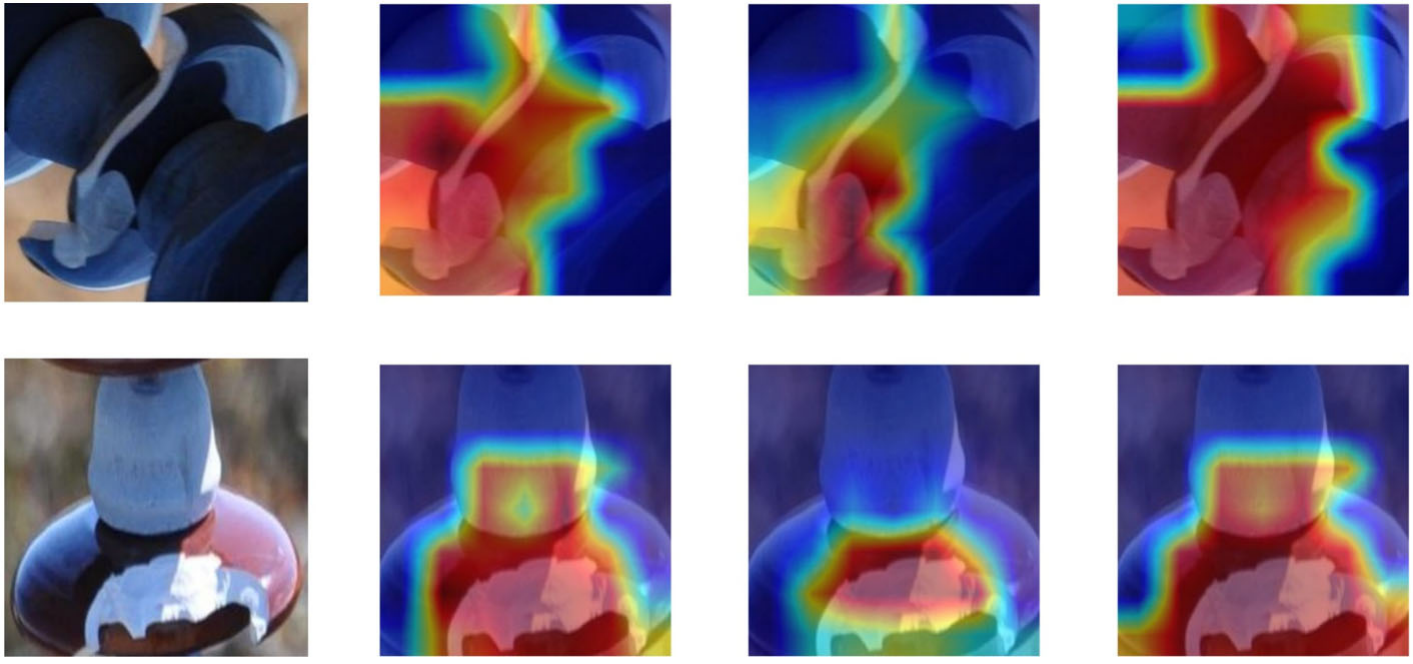
CAM results for broken insulators: The columns are the raw images, XGradCAM, HiResCAM, and ScoreCAM, respectively

**Figure 17:**
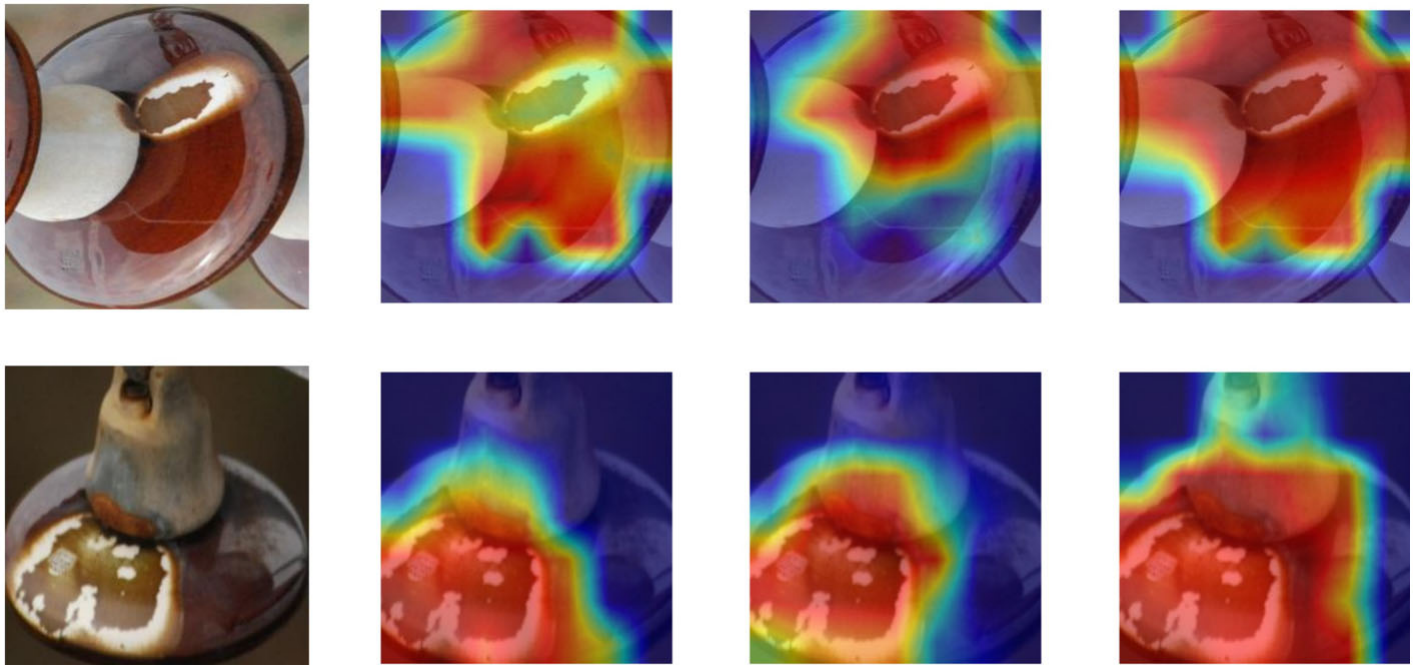
CAM results for flashover damage insulators: the columns are the raw images, XGradCAM, HiResCAM, and ScoreCAM, respectively

To address these challenges, eXplainable Artificial Intelligence (XAI) techniques have been adopted to provide insight into model behavior. Figure 16 (for broken insulators) and Fig. 17 (flashover damage insulators) present results of different CAM methods. In these figures, the CAM methods highlight in red the areas identified by the model as activation maps, thus creating this fault interpretability.

**Figure 18:**
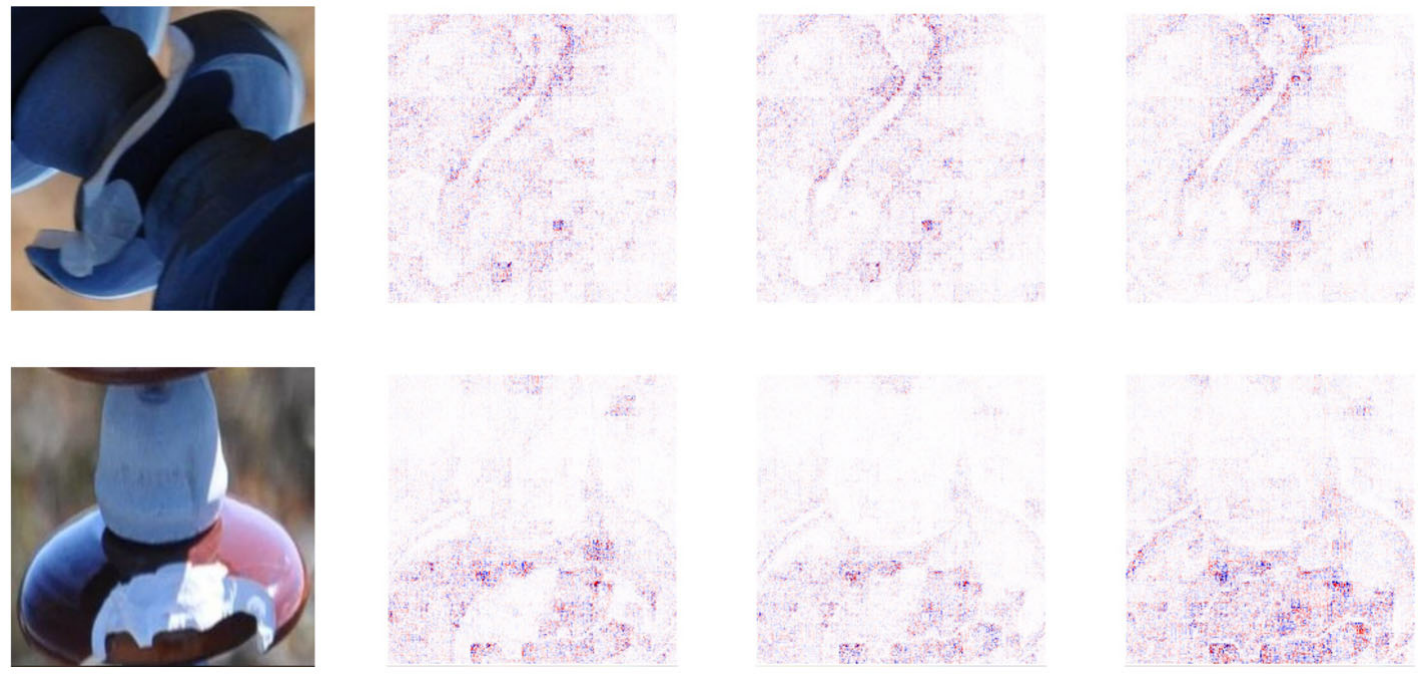
SHAP results for broken insulators: the columns are the raw images, the SHAP results for the broken class, the SHAP results for the flashover damage class, and the SHAP results for the no issues class

**Figure 19:**
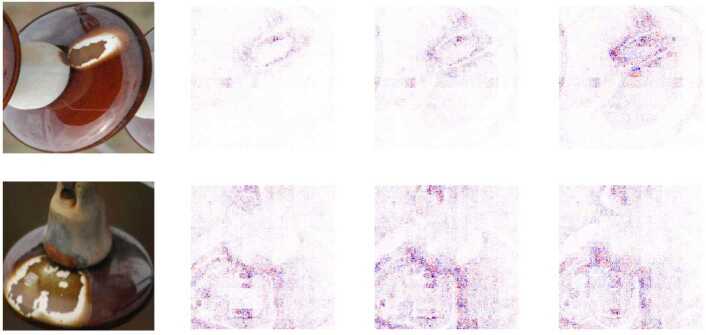
SHAP for flashover damage insulators: the columns are the raw images, the SHAP results for the broken class, the SHAP results for the flashover damage class and the SHAP results for the no issues class

Although the results are promising, the XGradCAM and ScoreCAM methods go beyond the limits of the fault by highlighting areas larger than the focus of analysis, so results using SHAP are evaluated. Figures 18 and 19 show the results obtained using SHAP. The blue results mean that they are features that move away from the class under study. Values coloured red mean that they are features that are closer to the class under study.

**Figure 20:**
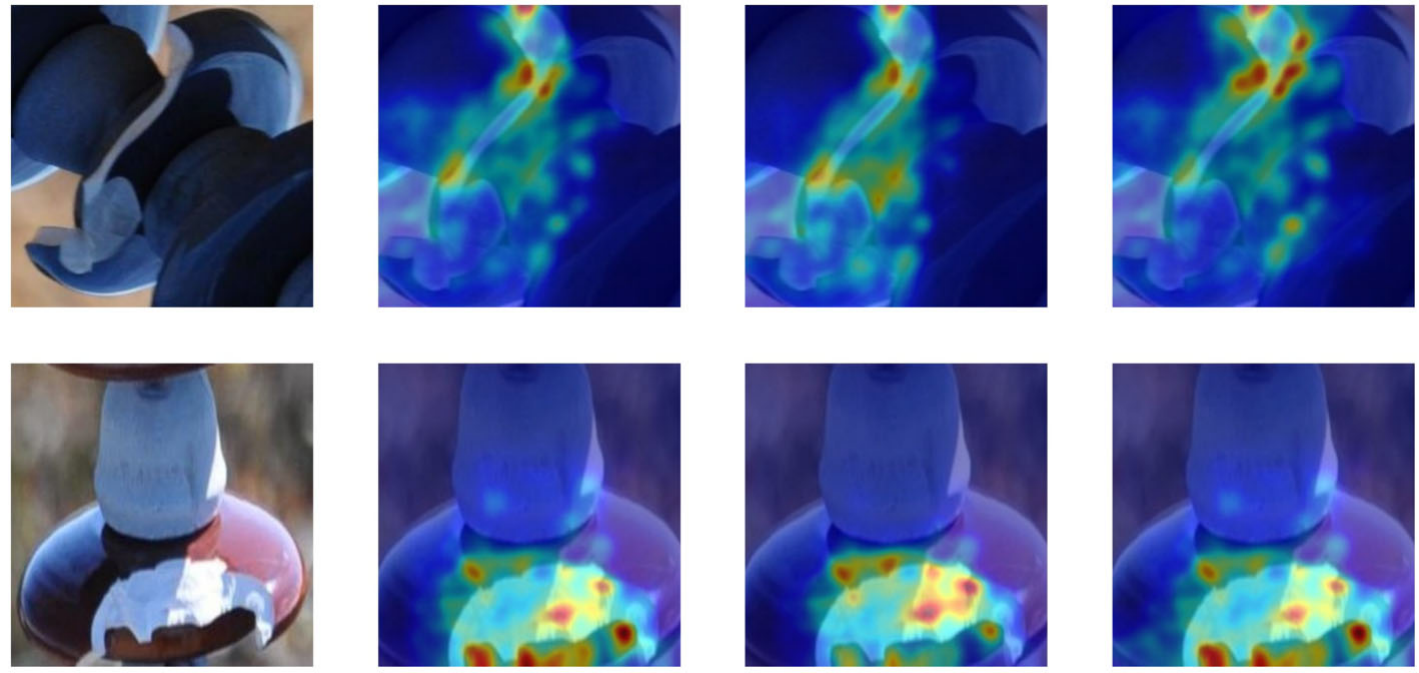
Proposed hybrid SHAP-CAM results for broken insulators: the columns are the raw images, SHAP-XGradCAM, SHAP-HiResCAM, and SHAP-ScoreCAM, respectively

**Figure 21:**
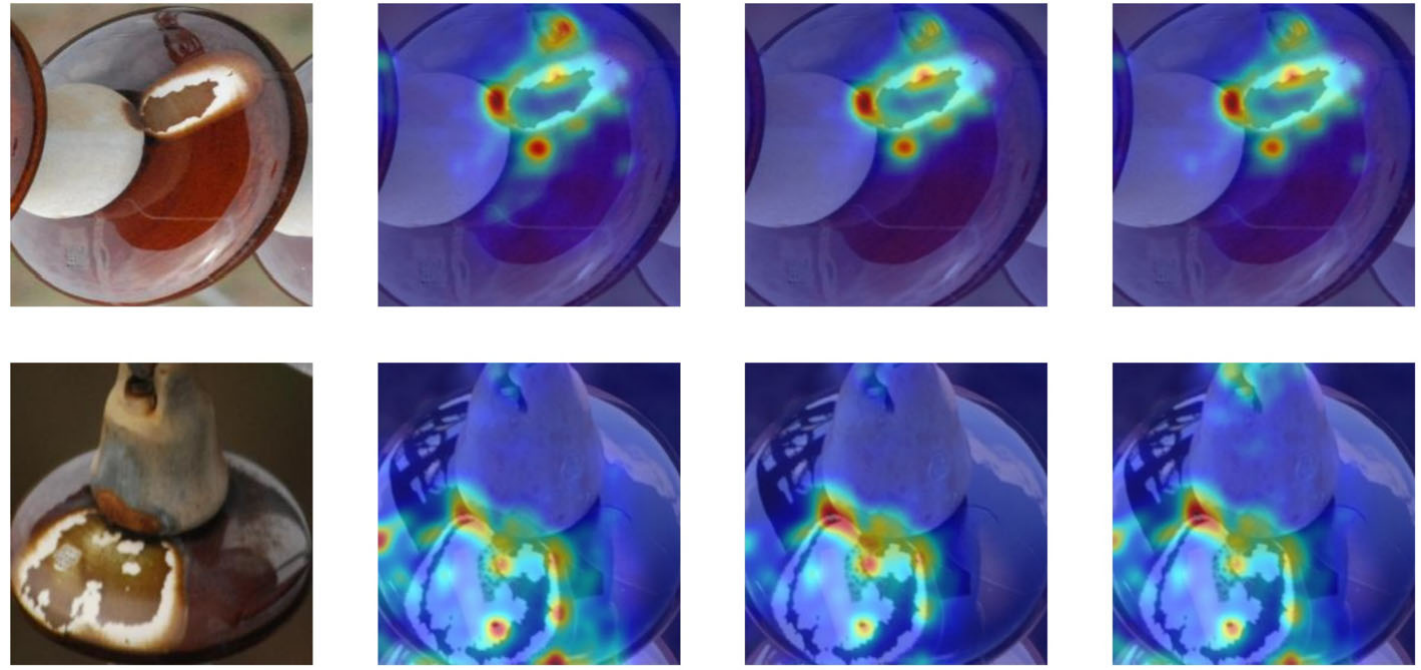
Proposed hybrid SHAP-CAM results for flashover damage insulators: the columns are the raw images, SHAP-XGradCAM, SHAP-HiResCAM, and SHAP-ScoreCAM, respectively

Based on the CAM and SHAP methods, this work proposed a combination of both to improve the visualization of the fault observed in the data provided. Figure 20 (broken insulator) and Fig. 21 (flashover damage class) show the results of this fusion, where visual improvement is noticeable and, for the most part, only the focus on the class to be detected is increased.

In the proposed hybrid SHAP-CAM method, there is greater clarity in highlighting the regions of evidence, which results in a better interpretation of the results. This hybrid SHAP-CAM method outperforms other methods, as it is possible to observe the exact location of the fault, rather than the large observation area as in other CAM methods, or the difficulty in interpreting SHAP.

The results are promising both when broken insulators are analyzed and when the evaluation is carried out on insulators with the presence of surface discharges (flashover). This highlighting indication can help operators to be more assertive in classifying faults when compared to good insulators or those that have small signs of contamination.

### Benchmarking

To carry out a comparative analysis, the DETR, Faster R-CNN, RetinaNet, SDD, YOLO (versions 10 to 24, sizes nano, small, medium, large, and extra large), STN-YOLO, and SAE-YOLO models are evaluated. These models are used to detect and classify the insulators simultaneously (multi-classification). Table 7 presents the performance results of this comparison; the default hyperparameters of each model are used so that our model can be compared with versions used in the literature.

**Table 7:** Insulator fault detection evaluation models’ prediction performance

Model	Precision	Recall	F1-score	mAP	
				[0.5]	[0.5:0.95]
DETR	0.69900	0.85619	0.76965	0.65116	0.46523
Faster R-CNN	0.41826	0.85230	0.56114	0.41211	0.26569
RetinaNet	0.27802	0.91727	0.42671	0.34637	0.23005
SSD	0.66123	0.58523	0.62091	0.32116	0.16968
STN-YOLO	0.90778	0.83811	0.87155	0.92821	0.78253
SAE-YOLO	0.88339	0.85990	0.87149	0.92579	0.79439
YOLOv10n	0.95897	0.95107	0.95452	0.97674	0.89722
YOLOv10s	0.95415	0.92386	0.93881	0.96839	0.90620
YOLOv10m	0.95047	0.95245	0.95177	0.97355	0.90680
YOLOv10l	0.94928	0.94527	0.94768	0.97151	0.90878
YOLOv10x	0.96555	0.94079	0.95315	0.97544	0.92064
YOLOv11n	0.97209	0.95530	0.96375	0.98379	0.90439
YOLOv11s	0.97219	0.95546	0.96376	0.98319	0.91813
YOLOv11m	0.97012	0.95904	0.96322	0.98219	0.92031
YOLOv11l	0.97068	0.96973	0.96996	0.98385	0.92990
YOLOv11x	0.96319	0.94591	0.95488	0.97249	0.89402
YOLOv12n	0.97189	0.95852	0.96474	0.97793	0.90716
YOLOv12s	0.96819	0.96520	0.96677	0.98173	0.92474
YOLOv12m	0.96348	0.96025	0.96258	0.97741	0.90668
YOLOv12l	0.95698	0.94239	0.95045	0.97730	0.91488
YOLOv12x	0.97624	0.94793	0.96201	0.97709	0.89832
YOLOv26n	0.97093	0.96164	0.96627	0.98018	0.91333
YOLOv26s	0.97028	0.95332	0.96173	0.98019	**0.93533**
YOLOv26m	0.97378	0.94267	0.95797	0.97497	0.92984
YOLOv26l	0.96329	0.96466	0.96398	0.97949	0.93174
YOLOv26x	0.96197	0.96491	0.96344	0.98340	0.93236
Ours	**0.98531**	**0.97797**	**0.98149**	**0.98951**	0.90891

Best results in bold

In general, the YOLO versions are more promising than other models, confirming their suitability for our baseline model. All standard versions of YOLO achieve an mAP@[0.5] greater than 0.96 and an mAP@[0.5:0.95] greater than 0.89, showing that they are indeed suitable for the task being analyzed. The simulation time can vary as the early stop is used to avoid overfitting the model, which means that training is stopped early to ensure that the model has been trained properly.

With an F1-score of 0.98149 and mAP@[0.5] of 0.98951, our method outperforms all compared models. In terms of computational effort, models that use a greater number of parameters have higher GFLOPs, which is not a limitation, considering that the inference time is acceptable for real-time applications.

Table 8 compares various models for insulator fault detection in terms of computational cost, training time, and inference speed. Classical detectors such as DETR, Faster R-CNN, RetinaNet, and SSD show moderate to high GFLOPs, with SSD achieving the fastest training (1.15 h) and DETR the lowest inference time (14.84 ms), though Faster R-CNN and RetinaNet are significantly slower during inference.

**Table 8:** Insulator fault detection evaluation of computational cost

Model	GFLOPs	Training time (h)	Inference time (ms)
DETR	234.96	2.15	14.84
Faster R-CNN	272.14	2.74	80.37
RetinaNet	245.77	2.86	65.41
SSD	91.28	**1.15**	22.09
STN-YOLO	10.70	7.06	5.51
SAE-YOLO	9.49	6.49	4.59
YOLOv10n	8.39	4.54	5.02
YOLOv10s	24.77	6.02	6.56
YOLOv10m	63.97	8.85	13.27
YOLOv10l	127.20	11.25	25.55
YOLOv10x	171.00	16.23	27.06
YOLOv11n	**6.44**	3.84	**3.78**
YOLOv11s	21.55	4.31	6.11
YOLOv11m	68.19	6.97	12.78
YOLOv11l	87.27	8.48	14.02
YOLOv11x	195.45	13.42	24.40
YOLOv12n	6.48	4.85	6.22
YOLOv12s	21.52	5.81	9.00
YOLOv12m	67.74	9.09	14.80
YOLOv12l	89.41	13.39	24.97
YOLOv12x	199.82	21.28	37.63
YOLOv26n	5.77	3.46	4.61
YOLOv26s	22.50	5.61	4.69
YOLOv26m	74.72	7.28	6.08
YOLOv26l	93.12	8.95	7.67
YOLOv26x	208.51	12.17	10.76
Ours	208.50	23.41	32.18

Best results in bold

The YOLO series demonstrates a clear trade-off between model size and efficiency, with YOLOv11n achieving the best balance of lowest GFLOPs (6.44) and fastest inference (3.78 ms), while larger variants of YOLOv11 and YOLOv12 increase computational cost and latency.

The proposed model exhibits high complexity (208.50 GFLOPs) and long training time (23.41 h, considering object detection and classification), yet maintains competitive inference speed (32.18 ms, considering object detection and classification), suggesting that it prioritizes detection performance and robustness over training efficiency. These results indicate that lightweight YOLO variants are optimal for real-time applications, whereas the proposed model suits scenarios where performance is more critical than training cost.

Although the full model integrates diffusion-based augmentation, transformer-based classification, and explainability modules, these components are primarily confined to the training phase, while the inference pipeline relies only on the hypertuned YOLO26x detector and the Swin-V2-B classifier operating on real images. Empirical results demonstrate that the detection stage achieves inference times on the order of a few milliseconds (e.g., 14.67 ms for YOLO26x), while classification requires approximately 17.51 ms, indicating that near real-time performance is already attainable on standard hardware.

The modular architecture enables straightforward lightweighting strategies, including replacing Swin-V2-B with smaller variants such as Swin-V2-T, reducing input resolution (e.g., 32×32 crops), and selecting compact YOLO variants (nano or small), which offer favorable trade-offs between performance and latency.

Additional optimizations such as model pruning, quantization, and edge-oriented deployment (e.g., TensorRT acceleration) can further reduce computational overhead. These results, combined with the demonstrated effectiveness of low-resolution inputs and smaller models, confirm that the proposed framework is adaptable to low-power UAV edge devices while preserving high detection and classification performance, supporting its practical viability in real-time engineering applications.

### Limitations

The conditional diffusion model needed 5.06 times longer to be trained than the conditional Wasserstein GAN to generate 256×256 images, which can be a problem for adaptive models. For images of 32×32, the conditional diffusion model needed 16.45 times longer to be trained than the conditional Wasserstein GAN. For offline applications such as those considered in this work, this limitation does not represent a problem since the model’s inference time is considerably faster once it has been trained.

## Conclusion and future research

This paper has presented a novel, end-to-end approach for fault detection in high-voltage insulators by integrating data augmentation, object detection, classification, and interpretability techniques. To address the challenge of the unbalanced dataset, the conditional diffusion model has been used to generate synthetic images of faulty insulators, achieving superior results compared to the conditional Wasserstein GAN. The model has enhanced transparency through a hybrid SHAP-CAM approach, providing intuitive visual explanations directly to field engineers, and helping the designers to better understand the strengths of the DL models.

The proposed YOLO-Swin architecture (hypertuned via Bayesian optimization) proved to have better results at both localizing insulators (mAP@[0.5] equal to 0.98951) and classifying fault conditions (F1-score=0.98149), outperforming the state-of-the-art detectors (DETR, Faster R-CNN, RetinaNet, SSD, YOLOv10–12, STN-YOLO, and SAE-YOLO) and CNN classifiers (VGG, ResNet, EfficientNet, ViT, Swin). Beyond these quantitative achievements, the proposed approach reduces the reliance on scarce real-fault images, enabling retraining to cover new insulator designs or damage conditions.

The evaluation under complex environmental conditions, such as rain, fog, and occlusion, remains an important direction for further validation of the proposed framework. However, the availability of publicly accessible datasets that comprehensively represent such challenging scenarios is currently limited, which restricts a systematic assessment in this context.

As future work, efforts could focus on incorporating more diverse and realistic environmental variations, either through the collection of new field data or through advanced data augmentation strategies, including physics-informed simulation and domain adaptation techniques. This would enable a more rigorous evaluation of model robustness and generalization capability under adverse inspection conditions, further strengthening the practical applicability of the proposed approach in real-world power grid environments.

To further strengthen the solution’s real-world applicability, future work may focus on expanding generative diversity by integrating multi-source weather and imaging conditions to capture a wider array of fault manifestations. Real-time deployment on edge devices, optimizing for latency and energy efficiency so that inspections can be performed via drones or on-site cameras. Adaptive learning through online fine-tuning allows the model to incorporate newly acquired fault instances and continuously improve detection performance. Cross-domain validation across different grid infrastructures and insulator materials to ensure broad generalizability and robustness.

## Data Availability

For future comparisons, the dataset is available at: https://github.com/jpmcarvalho/Optimized-YOLO.
